# Mechanistic stochastic model of histone modification pattern formation

**DOI:** 10.1186/1756-8935-7-30

**Published:** 2014-10-27

**Authors:** Lisette C M Anink-Groenen, Timo R Maarleveld, Pernette J Verschure, Frank J Bruggeman

**Affiliations:** Swammerdam Institute for Life Science (SILS), University of Amsterdam, Science Park 904, P.O. Box 94215, 1098 GE Amsterdam, The Netherlands; Systems Bioinformatics, Amsterdam Institute for Molecules Medicines and Systems, VU University Amsterdam, Amsterdam, The Netherlands; Life Sciences, Centrum Wiskunde & Informatica, Amsterdam, The Netherlands; BioSolar Cells, Wageningen, The Netherlands

**Keywords:** Chromatin structure, Histone modification patterns, Epigenetics, Stochastic mathematical model, Bistable dynamics, Boundary formation, Cooperative interactions

## Abstract

**Background:**

The activity of a single gene is influenced by the composition of the chromatin in which it is embedded. Nucleosome turnover, conformational dynamics, and covalent histone modifications each induce changes in the structure of chromatin and its affinity for regulatory proteins. The dynamics of histone modifications and the persistence of modification patterns for long periods are still largely unknown.

**Results:**

In this study, we present a stochastic mathematical model that describes the molecular mechanisms of histone modification pattern formation along a single gene, with non-phenomenological, physical parameters. We find that diffusion and recruitment properties of histone modifying enzymes together with chromatin connectivity allow for a rich repertoire of stochastic histone modification dynamics and pattern formation. We demonstrate that histone modification patterns at a single gene can be established or removed within a few minutes through diffusion and weak recruitment mechanisms of histone modification spreading. Moreover, we show that strong synergism between diffusion and weak recruitment mechanisms leads to nearly irreversible transitions in histone modification patterns providing stable patterns. In the absence of chromatin connectivity spontaneous and dynamic histone modification boundaries can be formed that are highly unstable, and spontaneous fluctuations cause them to diffuse randomly. Chromatin connectivity destabilizes this synergistic system and introduces bistability, illustrating state switching between opposing modification states of the model gene. The observed bistable long-range and localized pattern formation are critical effectors of gene expression regulation.

**Conclusion:**

This study illustrates how the cooperative interactions between regulatory proteins and the chromatin state generate complex stochastic dynamics of gene expression regulation.

**Electronic supplementary material:**

The online version of this article (doi:10.1186/1756-8935-7-30) contains supplementary material, which is available to authorized users.

## Background

Eukaryotic gene activity is strongly related to the epigenetic state [1–4]. Covalent histone modifications, chromatin folding into higher-order structures, and binding of large regulatory protein assemblies to the chromatin play a critical role in generating the epigenetic state. Changes in chromatin structure are guided by post-translational covalent histone modifications. Histones carry a multitude of posttranslational chemical modifications (for example, acetylation, methylation, ubiquitination, and phosphorylation) with dedicated patterns along the genome that correlate with defined gene expression patterns [2, 4, 5]. During differentiation, the large-scale chromatin structure and its histone modification composition defines whether the chromatin holds a permissive or restrictive chromatin state, which determines gene sensitivity to transcription factors; thereby establishing cell-type specific expression [2, 6].

Besides cell differentiation, covalent-histone modifications are proposed to mark transcription initiation, for example, involving ATP-dependent nucleosome remodeling, establishment of the pre-initiation complex, and allowing RNA polymerase II to start and proceed transcriptional elongation [7–14].

A prominent feature of core histones is the large amount and diversity of covalently modified residues they can possess [2, 15]. These marks guide the recruitment and binding of defined histone modifying enzymes and other regulatory proteins [16]. The histone modification composition along chromatin is often bordered by insulators, which likely demarcate the end of a local histone modification pattern [17, 18]. All of the above mentioned processes play a key role in regulation of transcription at the level of single genes [19–22].

A large body of evidence indicates that transcription regulation is dynamic and that it is inherently stochastic at various levels to give rise to large cell-to-cell heterogeneity [23] in transcriptional activity and in the resulting number of transcripts per cell [24]. The importance of the local epigenetic neighborhood of genes for stochastic transcription is becoming more apparent [21, 22, 25, 26]. The stochastic aspects of the establishment, maintenance, and decay of histone modification patterns are still largely unresolved. Some experiments indicate that the dynamics of these patterns may explain the large cell-to-cell variability of eukaryotic gene expression [22, 25, 27–29].

Insight into the mechanisms underlying the dynamics of histone modification pattern formation is largely lacking, mainly because many factors act in concert and simultaneous measurement or control of these factors is still experimentally challenging. In such a case, mathematical models can be effective to provide additional mechanistic insight into the molecular behavior of the system. Such models help to stimulate intuitive understanding and suggest new experiments to test the predicted behavior. Several computational efforts have enhanced our understanding of histone modification spreading and bistability [30–34]. For instance, the initial theoretical model of Dodd *et al.* [30] showed that the cooperativity of histones is essential to obtain bistability in distinct epigenetic states. However, the current body of computational models of histone modification patterning and spreading describes histone modification kinetics and cooperative processes using phenomenological process descriptions [30–36]. Although this is advantageous for reaching qualitative understanding of the dynamic modes of histone modification mechanisms, it does not allow us to predict the system consequences from basic biochemical and biophysical parameters. As a result, the exact time-scales of histone modification pattern formation and how protein-chromatin interactions and the movement of enzymes along the chromatin affect such patterns often remain unclear. Such knowledge is becoming more and more relevant as the field is moving in the direction of single-cell studies that address the influence of histone modification dynamics on gene regulation.

In this work, we study how the biochemical mechanisms of histone regulatory proteins give rise to spreading of histone modifications along the body of a gene [37]. We focus on the recruitment of regulatory proteins to chromatin [38, 39], the diffusion of regulatory proteins along the chromatin [40–42], and gene-connectivity through chromatin-chromatin interactions. To achieve this, we present a novel stochastic model with physical model parameters and mechanisms for chromatin binding, 1D diffusion, and recruitment of regulatory proteins, and gene connectivity based on biochemical mechanisms and literature-based kinetic parameters. This model is implemented in a dedicated software package that can be run as a plugin of the stochastic simulation software *StochPy* [43]. We study how this basic biochemical characterization of covalent histone modification turnover generates the establishment, maintenance, and decay of histone modification patterns along the body of a single gene. We find that histone modification patterns can be established within minutes by combined recruitment and diffusion mechanisms. A strong synergism between diffusion and weak recruitment leads to nearly irreversible transitions in histone modification patterns providing stable epigenetic patterns even in the absence of chromatin connectivity. In the absence of chromatin connectivity, spontaneous and dynamic histone modification boundaries can be formed but results in a highly unstable, fluctuation-driven state. Chromatin connectivity introduces bistability in the epigenetic state involving spontaneous, erratic switching between the two opposite (that is, a fully active and inactive) histone modification states of the model gene, and the formation of stable epigenetic histone modification patterns along the entire body of the gene. We demonstrate that such behavior can be found within biologically plausible parameter regimes. This study suggests an important role for epigenetics in gene activity regulation. The stochastic switching of the epigenetic state that we observe can contribute to cell-to-cell heterogeneity of transcription while the observed formation of a stable epigenetic pattern could play a role in robust gene silencing.

## Results

### The histone modification spreading model

**Figure 1:**
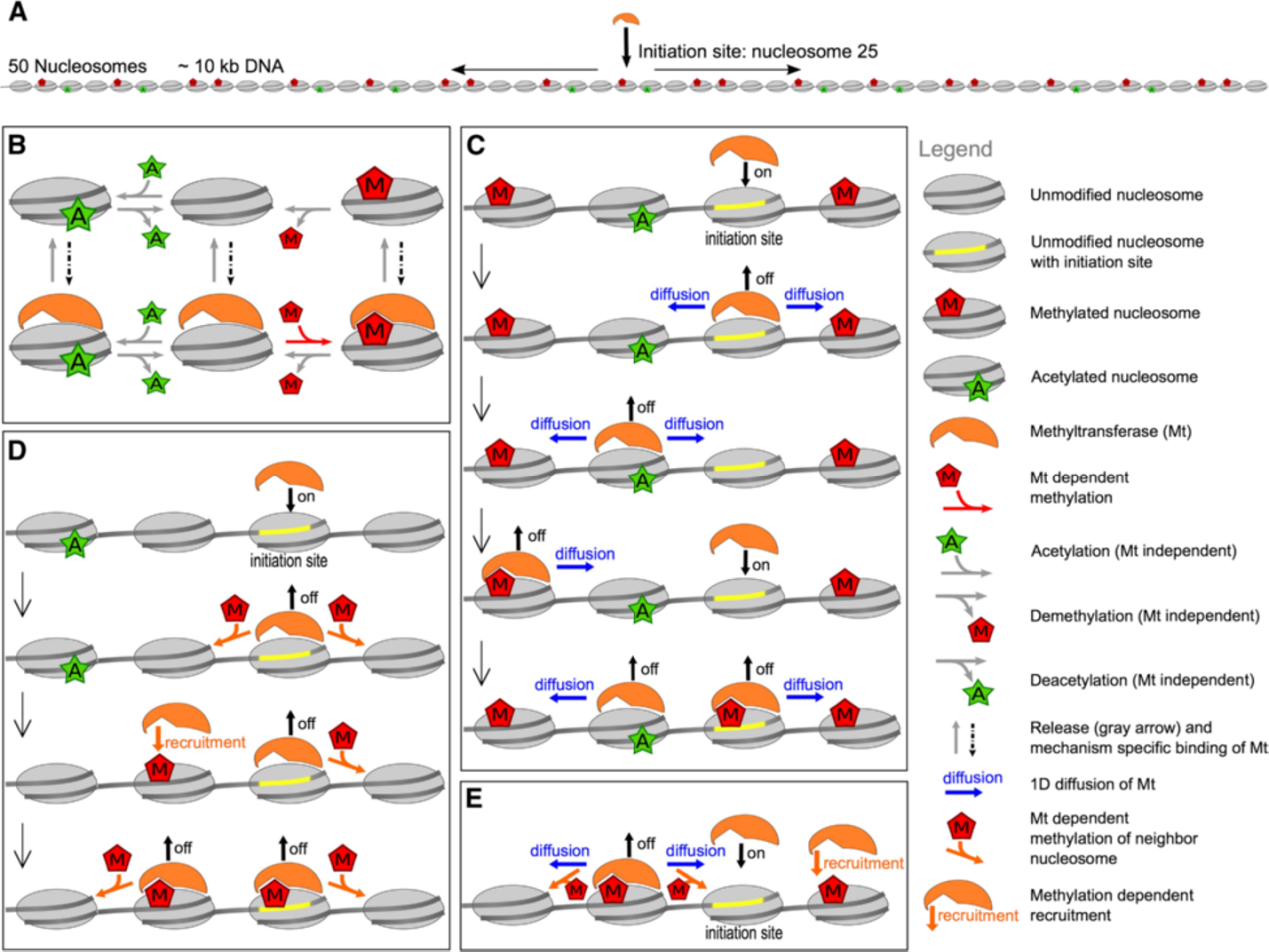
**Building blocks of the histone modification spreading and pattern formation model. (A)** The nucleosome model consists of an array of 50 nucleosomes with one specific initiation site for the methyl- and acetyltransferases (indicated by the arrow). In **(B)** the nucleosome modification reactions are depicted, while **(C-E)** indicate the binding and movement properties of the transferase. **(B)** The nucleosomes can be in three modification states: A (acetylated; green star), U (unmodified), or M (methylated; red pentagram). The methyltransferase can bind to a nucleosome in any modification state but can catalyze only the reaction from U to M. Both the acetylation reactions, the conversion of U to A, and the demodification reactions from both A and M to U are ubiquitous and independent of the presence of the methyltransferase. Dashed arrows represent the binding of the transferase to the nucleosome. Binding of a transferase to a nucleosome occurs either by binding at the initiation site, by 1D diffusion, or by the recruitment process. **(C)** 1D diffusion of the transferase over the array in hops of one nucleosome. The transferase exclusively enters the nucleosome array at the specific initiation site (yellow) and has at each position equal chance to move left or right. Both the binding and diffusion reactions are independent of the modification state of the nucleosomes. The transferases cannot move over a neighboring transferase or a boundary. **(D)** Modification-induced transferase recruitment. The transferase lands on the initiation site (yellow) independent of the modification state of the nucleosome and can methylate the bound nucleosome and its neighbors. Methylated nucleosomes can recruit additional transferases. **(E)** Combined diffusion and modification-induced transferase recruitment. This mechanism combines all the characteristics of the two single movement mechanisms. Reactions are given in Additional file 10: S5.

We consider a nucleosomal chromatin region of 10 kb corresponding to the length of an average human gene (Figure 1A). In the model this chromatin region is represented by an array of 50 nucleosomes, as a single nucleosome covers 147 bp of DNA and about 50 bp in linker DNA. Nucleosomes consist of histone octamers containing two copies of each histone H2A, H2B, H3, and H4. The tails and globular domains of the histones can be enzymatically modified at various sites [2, 15]. To reduce this biochemical complexity to its essence, we treat each of the nucleosomes as a single unit that can exist in only one out of three modification states: acetylated (A), unmodified (U), or methylated (M; Figure 1B). We use these modifications as examples for active, neutral, and silenced chromatin. We refer to the histone modifications as nucleosome modifications or simply as modifications. These enzymes are considered to catalyze the methylation and acetylation reactions. From here on we will refer to these enzymes as the methyltransferases and acetyltransferases. The model considers explicitly the binding, 1D diffusion, and recruitment of the transferases. We assume that the methyl- and acetyltransferase can only modify the nucleosome in its unmodified state (from U to M or U to A), and that each nucleosome can be bound by only one transferase at a time (Figure 1B). The conversions from a modified to unmodified state are assumed to exhibit basal activity of the demodification enzymes; in other words, we do not track the binding, diffusion, and recruitment of the demodification enzymes.

In the simulations, the modification process is initiated from a specific site in the nucleosomal array. This targeted nucleosome either corresponds to a nucleosome with a specific histone modification composition, or to a nucleosome-free region (for example, a 3’ or 5’-NFR) with an accessible protein-DNA binding domain. Such 5’-NFRs often demarcate the transcription start site. They are typically enriched in transcription factor binding sequences [44] and known to be involved in the induction of transcription [22, 45]. Here, we assume this initiation site or DNA binding site at the position of one designated nucleosome in the center of the array and we refer to this nucleosome as the initiation site (Figure 1A).

First, we monitor only the methyltransferase explicitly and we assume the acetyltransferase as background activity. The modification mechanism of a single nucleosome is given in Figure 1B. The choice for the methyltransferase here is arbitrary, as the reverse situation - that is, an explicit acetyltransferase and background methyltransferase - would give identical results with the selected parameters. Therefore, conclusions that are drawn from our model simulations can be applied to either activation or silencing of the modeled gene. Later we relax this assumption and track the fate of both the acetyl- and the methyltransferase, and add ‘jumping’ of the transferase by means of chromatin connectivity.

We distinguish several mechanisms for transferase movement along the nucleosomal array: diffusion (Figure 1C), modification-induced recruitment (Figure 1D), and a combination of both (Figure 1E). Transferase diffusion is based on the findings that proteins can display 1D diffusion along DNA and chromatin [40, 46–49], which is implemented in the model as a 1D random walk. A diffusion-event is reflected in the simulations as a discrete step from one nucleosome to a neighboring nucleosome (Figure 1C). Note that when a transferase encounters another transferase on the neighboring nucleosome it cannot push or diffuse over this obstacle. Before a next diffusion step, the transferase can modify an encountered unmodified nucleosome. The next mechanism, modification-induced recruitment (Figure 1D), corresponds to a mechanism observed during the formation of heterochromatin induced by Heterochromatin Protein 1 (HP1) (reviewed by [11]). HP1 binds with its chromo domain to chromatin at histone H3 trimethylated at lysine 9 (H3K9me3) [38]. With its chromo shadow domain HP1 can homodimerize with HP1 isoforms or heterodimerize with histone methyltransferase enzymes such as SUV39h1 (the mammalian ortholog of *Drosophila* suppressor of variegation 3-9 (Su(var)3-9)), thereby inducing spreading of H3K9Me3 [50]. Here, we simplify the properties of HP1 and SUV39h1 (histone modification binding and methylation of neighboring histones) into properties of the single transferase in our model. Thus, a methyltransferase with modification-induced recruitment properties can bind at any methylated nucleosome, and it can methylate both the occupied nucleosome and neighboring nucleosomes. However, the methyltransferase will not diffuse between the nucleosomes. In this sense the mechanism is similar to previously published models [30, 31]. In a model with combined diffusion and recruitment properties (Figure 1E) the transferase can perform all of these activities: diffuse to a neighboring nucleosome, bind to a methylated nucleosome, methylate the bound nucleosome, and methylate a neighboring nucleosome. Regardless of the propagation mechanism, the transferase can dissociate from a nucleosome at any time and position.

The boundaries of the nucleosomal model array are reflective, thus a transferase at the border perceives the boundary as another nucleosome that is occupied by another transferase. The transferase at the boundary can either dissociate from the nucleosome or move away from the boundary. The kinetic parameters of the model can be found in Table 1 and their derivation is described in the Methods section. All the mathematical models are stochastically simulated with a dedicated NucleosomeTool plug-in for the software package *StochPy (Stochastic modeling in Python)* [43], which uses the Gillespie algorithm for stochastic simulations (see Methods). This plug-in consists of a NucleosomeModelBuilder, that builds the models used in this article, and a NucleosomeSimulator, that handles the stochastic simulations and analysis of these models. Usage of the NucleosomeTool plug-in is described in the Additional file 1: S1 of this paper together with the scripts to run the models (Additional file 2: S2, Additional file 3: S3 and Additional file 4: S4).

**Figure 2:**
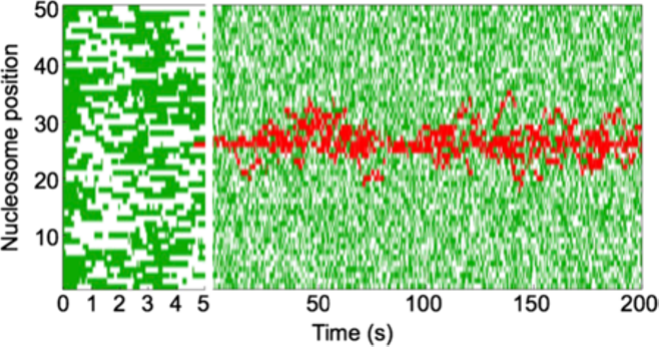
**Example simulation of the 1D diffusion transferase mechanism.** Over a 200-s period, the modification state of each nucleosome is recorded with the acetylated state in green and methylated state in red. The initiation site is situated at position 25, this is the only point of influx of the methyltransferase. The first 5 s of the simulation are shown in higher detail. Parameters used in this simulation are given in Table 1 with *k*_*on*_ = 2.4 s^-1^ .

As an illustration, we show in Figure 2 a simulation of the model using a transferase with diffusion properties and with its initiation site at the center of the nucleosomal array. Initially, we start with 50 acetylated nucleosomes and then allow a methyltransferase to bind its initiation site. The initial 50 acetylated nucleosomes are deacetylated and acetylated rapidly, and form a dynamic background in which the nucleosome is in an unmodified state half of the time. The diffusing transferase introduces a time-dependent histone modification pattern.

**Table 1:** **Overview of the kinetic parameters in the model**

Parameter	Description	Value (s ^-1^)
*k*_*on*_	Influx of transferases at initiation site	2.4 (one enzyme models)
		0.01 (two enzyme models)
*k*_*off*_	Release rate of transferase from nucleosome	0.1
*k*_*transferase*_	Modification rate of nucleosome	1000
*k*_*neighbor*_	Modification rate of neighboring nucleosome	0.2
*k*_*slide*_	1D diffusion rate over the chromatin	0.6
*k*_*recruitment*_	Influx of transferases at modified nucleosome	0.24-4.8
*k*_*demodification*_	Rate constant of demodification	2.4
*k*_*interaction*_	Interaction frequency	0.01-1

The derivation and calculations of the kinetic parameters can be found in the Methods section.

### 1D diffusion and recruitment mechanisms show localized modification patterns

We introduce four characteristics to analyze how modification patterns are established, maintained, and removed with the three mechanisms of transferase movement. The first characteristic is the stationary methylation and methyltransferase occupancy pattern along the array in steady state. Such a simulated stationary pattern corresponds to a histone modification pattern as observed with ChIP-seq analysis. The second characteristic is the activation dynamics. This characteristic is plotted as the methyltransferase occupancy on the nucleosomal array starting from the fully acetylated array state. As third characteristic we compute the steady-state probability distribution of the number of methyltransferases and the three modification states on the array. Finally, we test the relaxation dynamics. This fourth characteristic determines the stability of a modification pattern. To test this we simulate the system from an initial state in which all nucleosomes are methylated and bound to a methyltransferase, subsequently, we determine the decline in the number of methylated nucleosomes. In this fourth test we do not allow any influx of new methyltransferases at the initiation site and we assume that the initiation site is inaccessible, for instance due to nucleosomal repositioning.

**Figure 3:**
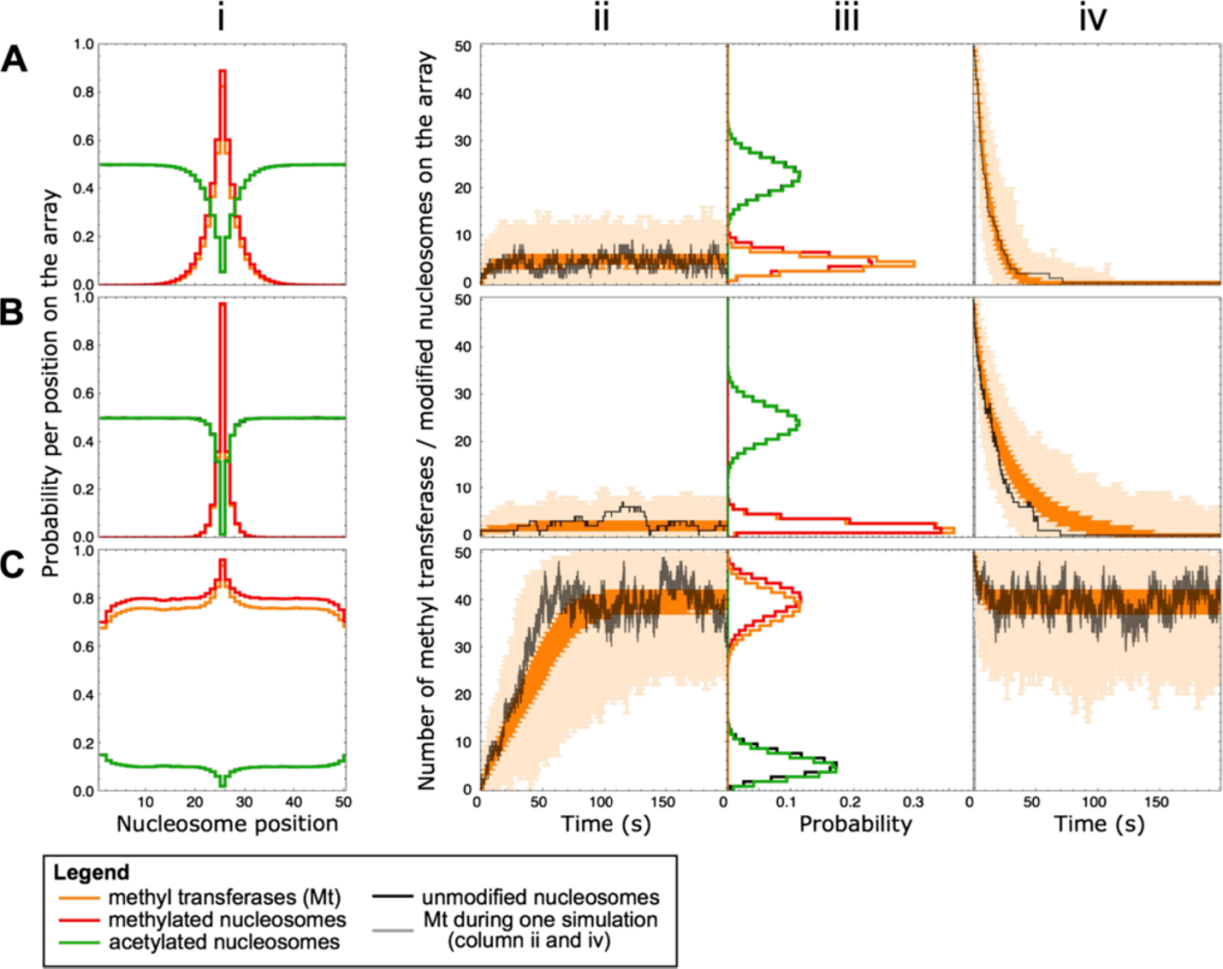
**Performance of three histone modification spreading mechanisms.** The rows show data for the **(A)** diffusion, **(B)** modification induced recruitment, and **(C)** combined diffusion and recruitment mechanisms. **(A, B, C)** Column i shows the model-predicted stationary histone modification patterns. The y-axis indicates the probability per nucleosome to be modified or occupied by a methyltransferase (Mt). Red: methylation mark, orange: methyltransferase, green: acetylation mark, and black: unmodified state. In the second column (labeled ii) the time to establish a stationary methylation state is evaluated. Simulations started from a condition in which all the nucleosomes are in the A state. The dark orange error bars contain 50% of the data points around the median and the light orange error bars show the minimal/maximal value regarding the number of methylated nucleosomes (binned/second). An example trace of a single simulation is shown in black. In the third column (iii) the probability distribution of the total transferase number and total modifications at steady state are displayed (same color coding as in column i). Column iv displays the relaxation dynamics. The error bars in column iv have the same meaning as in column ii. The simulations are initiated with all nucleosomes in the methylated state and occupied with methyltransferases, influx of new methyltransferase is not allowed at the initiation site. Parameters for all simulations are given in Table 1, with *k*_*on*_ =2.4 s^-1^ and in (B, C) *k*_*recruitment*_ =2.4 s^-1^.

**Figure 4:**
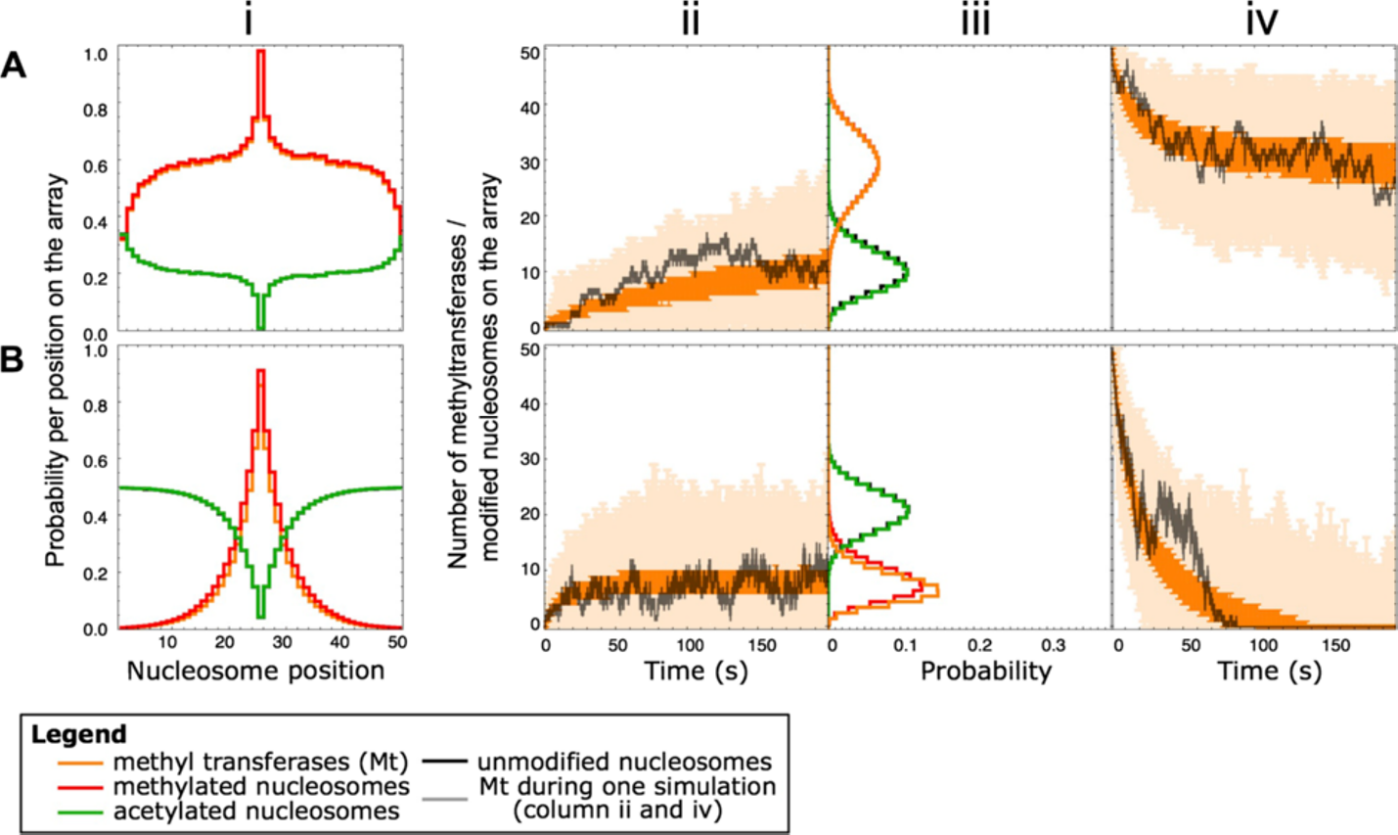
**Performance of recruitment and combined spreading mechanisms with adjusted RE.** The rows show data for the **(A)** modification induced recruitment mechanism, RE = 2, *k*_*recruitment*_ = 4.8 s^-1^ and **(B)** combined diffusion and recruitment mechanism, RE = 0.1, *k*_*recruitment*_ = 0.24 s^-1^. The other parameters used in these simulations are listed in Table 1. **(A, B)** In column i the stationary histone modification patterns as predicted by the model are shown. The y-axis indicates the probability per nucleosome to be modified or occupied by a methyltransferase (Mt). Red: methylation mark, orange: methyltransferase, green: acetylation mark, and black: unmodified state. In the second column (labeled ii), the time to establish a stationary state of methylation on the gene is displayed. Simulations started from a condition in which all the nucleosomes are in the A state. The dark orange error bars contain 50% of the data points around the median and the light orange error bars show the minimal and maximal value of the number of methylated nucleosomes (binned per second). An example trace of a single simulation is shown in black. In the third column (labeled iii), the probability distribution of the total transferase number and the total modification amount at steady state are displayed (same color coding as in column i). Column iv displays the relaxation dynamics. The error bars in column iv have the same meaning as in column ii. The simulations are initiated with all nucleosomes in the methylated state and occupied with methyltransferases, influx of new methyltransferase is not allowed at the initiation site.

In Figure 3, we analyze the diffusion, recruitment, and the combined diffusion and recruitment mechanisms on these four characteristics. Comparison of the characteristics of the transferase diffusion mechanism and the modification-induced recruitment shows that these mechanisms give remarkably similar output (Figure 3A and 3B; Figure 3A has the same parameters as Figure 2, listed in Table 1). Both mechanisms produce a small-width peak across the initiation site (Figure 3Ai and 3Bi). The stationary level of methyltransferases is in both cases reached in seconds (Figure 3Aii and 3Bii), and the average level of methyltransferases and methylation on the array is relatively low (Figure 3Aiii and 3Biii). Furthermore, both mechanisms are incapable of maintaining the obtained pattern in the absence of the initiation signal (Figure 3Aiv and 3Biv). In the recruitment mechanism, we considered a relatively low recruitment-efficiency (RE) of 0.5. The RE is defined as the lifetime of the modification divided by the average time before a recruitment event. Thus if the lifetime of the modification is short compared to the time needed for recruitment of a transferase, the RE is low. In Figure 4A, we show that increasing the RE (from 0.5 to 2) can increase the stationary methylation state and the stability of this state. Increased transferase recruitment can, for instance, be induced by an enhanced affinity of the methyltransferase for the methylated nucleosomes. In the case of HP1-induced silencing, increased transferase recruitment could be caused by an increased affinity of HP1 for a specific combination of histone modifications on one nucleosome. This suggests that local histone modification combinations could be able to tune the longevity and stability of long-range histone modification patterns. These results indicate that diffusion and low RE recruitment mechanisms give rise to modification patterns that have been observed at transcription start sites and promoters in genome-wide ChIP-seq studies [2, 5, 9, 20]. In this simulation, the methyltransferase would have to be replaced by the acetyltransferase to establish active modification patterns as noticed experimentally on promoter and enhancer sites. Moreover, similar modification patterns are created upon simulating either a methyltransferase or acetyltransferase.

### Strong synergism between 1D diffusion and recruitment leads to bistable behavior

In the combined diffusion and recruitment mechanism, the parameters for diffusion and recruitment of the separate mechanisms are combined. Here, the number of methyltransferases on the array at steady state increases drastically (Figure 3Ci). Separately, the diffusion and recruitment mechanisms generate a local modification pattern around the initiation site, while the combination of diffusion with recruitment produces a widely spread histone modification pattern which peaks at the initiation site and covers the entire array. This indicates that diffusion and low RE recruitment have a strong synergistic relationship. The combined mechanism gives rise to a methylation pattern that spreads across the body of the gene, which is also experimentally observed in ChIP-seq studies and has a resemblance to widespread heterochromatin methylation patterns [2, 9, 20]. The observed lower probability of methylation at the boundaries of the array (Figure 3Ci) is the result of a lack of transferase influx from ‘outside’ the boundaries. Interestingly, this elevated methylation state obtained with the combined mechanism is reached within minutes (Figure 3Cii). The probability distributions for the methyltransferase and the three modification states in steady state are shown in Figure 3Ciii. The dispersion in these distributions is quite small.

The relaxation dynamics of the combined mechanism shows an unanticipated result (Figure 3Civ). In contrast to the instability of the diffusion and recruitment mechanisms, the methylation pattern across the array is maintained while influx at the initiation site is ceased. This shows that the recruitment influx of methyltransferases can compensate for the lack of initiation events at the initiation site, even though, this recruitment process by itself is inefficient and does not give rise to a long-lived pattern (Figure 3Biv). The stability of the methylation pattern in the combined mechanism can be reduced when the RE is reduced (Figure 4B). As shown in Figure 4A, increased transferase recruitment in the recruitment mechanism causes increased spreading of the modification pattern and a stable steady state in the absence of influx of transferases. However, this high-level RE recruitment mechanism shows appreciably slower dynamics than is observed using the combined mechanism. The stability of the epigenetic state in the absence of an initiation signal is a prerequisite for long-range silencing in developmental genes. Therefore, this combined diffusion and recruitment might represent a mechanism to induce long-range heterochromatic silencing.

**Figure 5:**
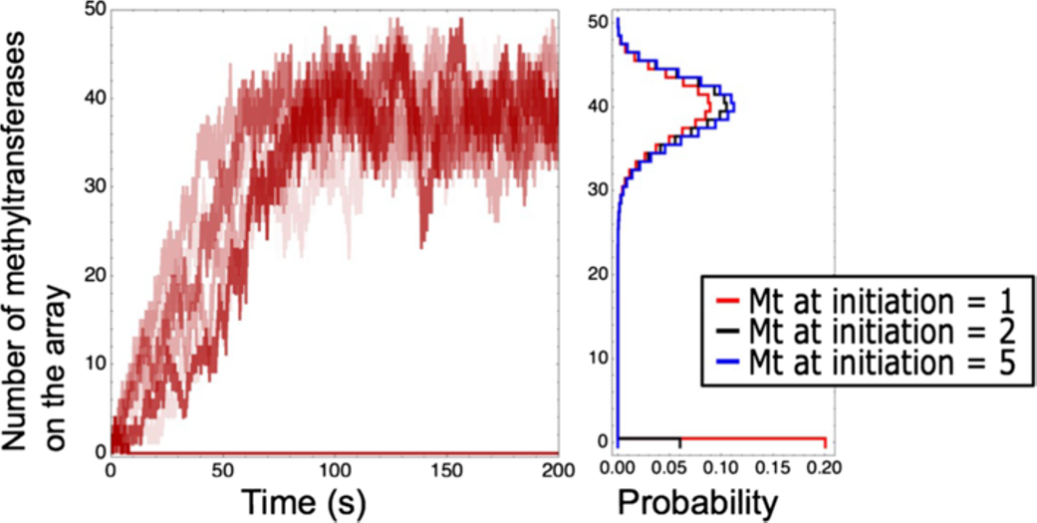
**Analysis of the minimally required methyltransferases for stationary methylation pattern formation with the combined mechanism.** Equal parameters are used as in Figure 3Civ only the initial methyltransferase occupation is lowered from 50 to 5, 2, and 1 equidistantly positioned methyltransferases. The left panel shows 10 example simulations of which 8 end in a high methylation state and 2 in a state without methylation. The right panel shows bimodal steady-state distributions of the system with 1, 2, and 5 initial methyltransferases. The steady-state distribution of the number of methyltransferases is shown. The parameters used in these simulations are listed in Table 1, with *k*_*on*_ = 2.4 s^-1^ and *k*_*recruitment*_ = 2.4 s^-1^.

We additionally investigated the amount of methyltransferases that are minimally required as initial ‘seeds’ for the induction of a stationary modification pattern in the combined mechanism. We observed in simulations that started with a single methylated nucleosome occupied by a methyltransferase that, in 80% of the 400 simulations (Figure 5) a stable methylation pattern of approximately 40 methylated nucleosomes was induced (equal to the final state of Figure 3Ciii). The remaining 20% of the simulations end in a stationary state without any methylated nucleosomes. Simulations that start with two or five methylated and occupied nucleosomes reach the stationary state of approximately 40 methylated nucleosomes in respectively 94% or 100% of the simulations (Figure 5). These data indicate that a form of stochastic bistability exists, depending on the initial number of methylated sites the system exhibits a probability to reach a stationary state demonstrating a methylation pattern or a state without this pattern. The probability to reach such a methylated state increases with the number of seed sites. This confirms that the combination of the diffusion and recruitment mechanism provides extreme synergy and potency.

### Introduction of two opposite modification enzymes causes unstable boundary formation

**Figure 6:**
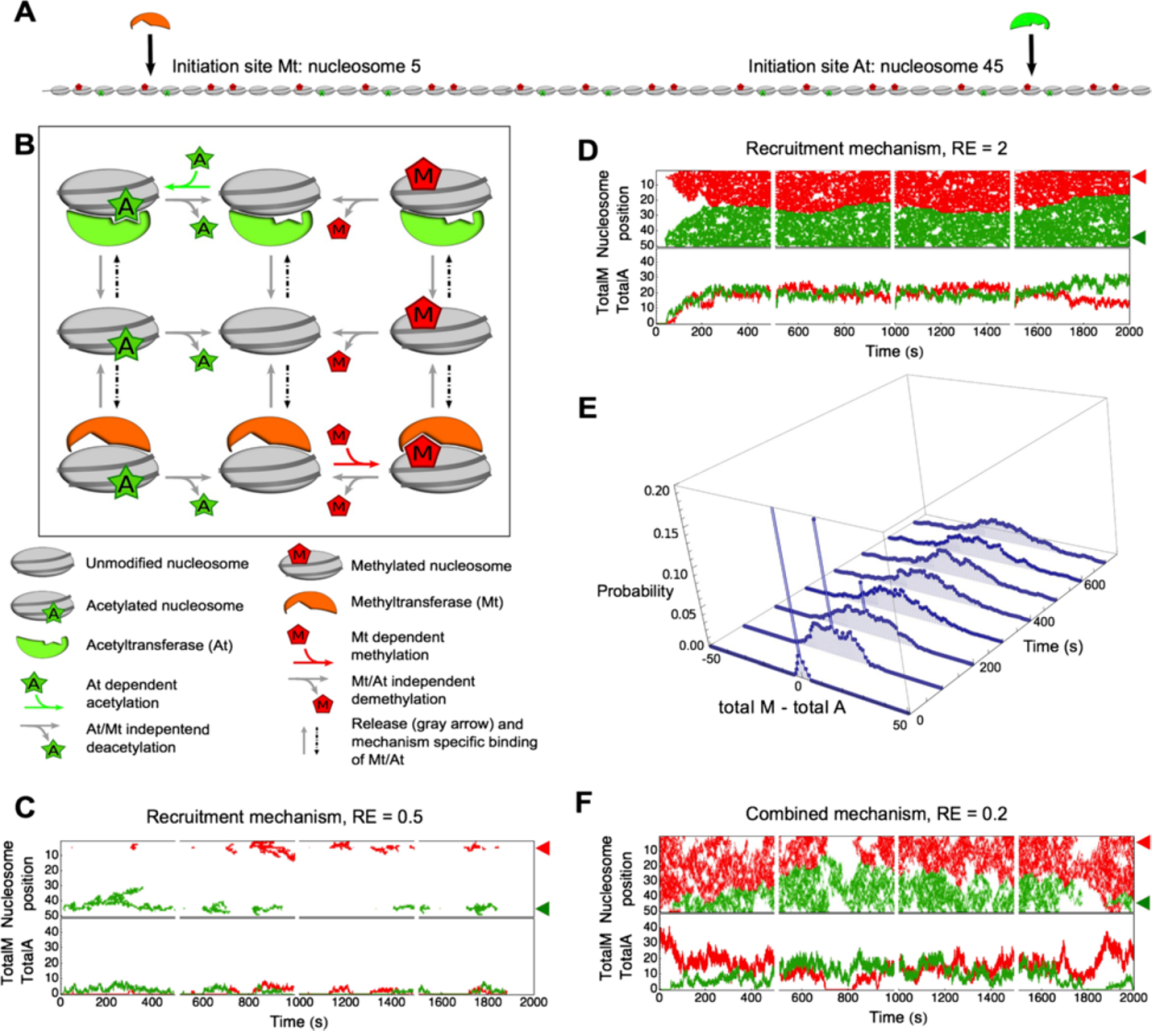
**Characteristics of opposing transferases and formation of a modification boundary. (A)** On the nucleosomal array consisting of 50 nucleosomes both the methyltransferase (Mt) and acetyltransferase (At) have an initiation site, at nucleosome position 5 and 45, respectively. **(B)** With the introduction of the acetyltransferase to the system the acetylation reaction is changed into a transferase dependent reaction. The demodification reactions both remain transferase independent. Dashed arrows represent the binding of the transferase to the nucleosome. Transferase nucleosome binding is caused by transferase binding at the initiation site, by diffusion, or by the recruitment process (Figure 1C-E). Two transferases cannot bind to one nucleosome at the same time. A full list of model reactions can be found in Additional file 10: S5. **(C-E)** Each figure shows four simulations of 500 s as an illustration of the model behavior. Top panels show the position of the methylation (red) and acetylation (green) over time, initiation sites are indicated by red and green arrowheads. Bottom panels show the total amount of modifications on the array over time, corresponding to the top panels. The parameters used in these simulations are listed in Table 1, with *k*_*on*_ = 0.1 s^-1^. Model behavior is shown for **(C)** the recruitment mechanism with low recruitment-efficiency (RE = 0.5; *k*_*recruitment*_ =1.2 s^-1^) and **(D)** recruitment mechanism with high recruitment-efficiency (RE =2; *k*_*recruitment*_ = 4.8 s^-1^). **(E)** This figure shows the boundary behavior of the recruitment mechanism shown in **(D)**. Within intervals of 10 s the distributions of total methylated nucleosomes minus total acetylated nucleosomes is calculated over 450 simulations. Probability distributions of the intervals starting at 0 s, 100 s, 200 s, 300 s, 400 s, 500 s, 600 s, and 700 s are shown. **(F)** This figure shows model behavior of combined recruitment and diffusion mechanism with low recruitment-efficiency (RE = 0.2; *k*_*recruitment*_ =0.48 s^-1^).

In the previous section, we studied the response properties and patterns of histone modifications by tracking only the methyltransferase and assuming that the antagonistic enzyme (the acetyltransferase) is active in the background. Here we extend our model. We additionally track the acetyltransferase explicitly such that the methyl- and acetyltransferase compete for nucleosome binding. Our parameter settings change such that the two opposite transferases have equal diffusion and recruitment properties and that they each have their own initiation site, at nucleosome position 5 and 45 for the methyl- and acetyltransferase, respectively (Figure 6A). The methyl- and acetyltransferase are unable to occupy the same nucleosome at the same time or to ‘hop’ over each other (Figure 6B). The demethylase and deacetylase remain ubiquitous background reactions.

Simulations performed with a single transferase indicated that an elevated influx of transferases at the initiation site causes a nearly permanent occupation of the initiation site. This situation leads to stable modification peaks around each initiation site (Figure 3 column i). Because we are interested in the origin of bistability we study conditions in which one modification overtakes the other. Therefore, we lower association rates at the initiation site, and take a rate constant of 0.01 s^-1^ for the initiation rate (k_initiation_).

The recruitment mechanism with RE = 0.5 (so a low initiation rate) yields a modification pattern close to the initiation sites (Figure 6C). This is in agreement with Figure 3B in which each of the transferases cannot modify the nucleosomes further away from their initiation site. When the transferases are simulated with a higher efficiency recruitment mechanism (RE = 2.0) the opposing transferases form a ‘boundary’ in the center of the nucleosomal array (Figure 6D). In this case, the transferases can reach nucleosomes far from their initiation sites but since they are not able to pass each other, a clear boundary between acetylation and methylation states is generated. This boundary is on average in the middle of the array but its location is unstable. Moreover, methylation and acetylation can outcompete each other to give rise to a nucleosomal array that is either totally methylated or acetylated. The initiation site of the methyltransferase or acetyltransferase can be blocked by the presence of an antagonistic transferase on the initiation site. New influx of transferases is hampered if the k_recruitment_ is higher than k_initiation_ and as a result, the entire system remains mostly in one modification state. When an initiation event occurs, in principle, the modification that is outnumbered is able to overtake the opposite modification but the probability of this event is relatively low. This simulation, therefore, indicates that spontaneous boundary formation by opposing modifications may eventually lead to one modification dictating the opposite modification. In Figure 6E, an indication of the stability of the boundary position is given by the difference between total methylation and total acetylation on the array. Within the 700 s of simulated time the recruitment mechanism rarely causes the formation of states where the majority of the array is methylated or acetylated. The combined recruitment and diffusion mechanism with RE = 0.2 (Figure 6F) also shows this boundary formation although in a more dynamic fashion.

### Chromatin connectivity induces bistable dynamics in modification pattern formation

**Figure 7:**
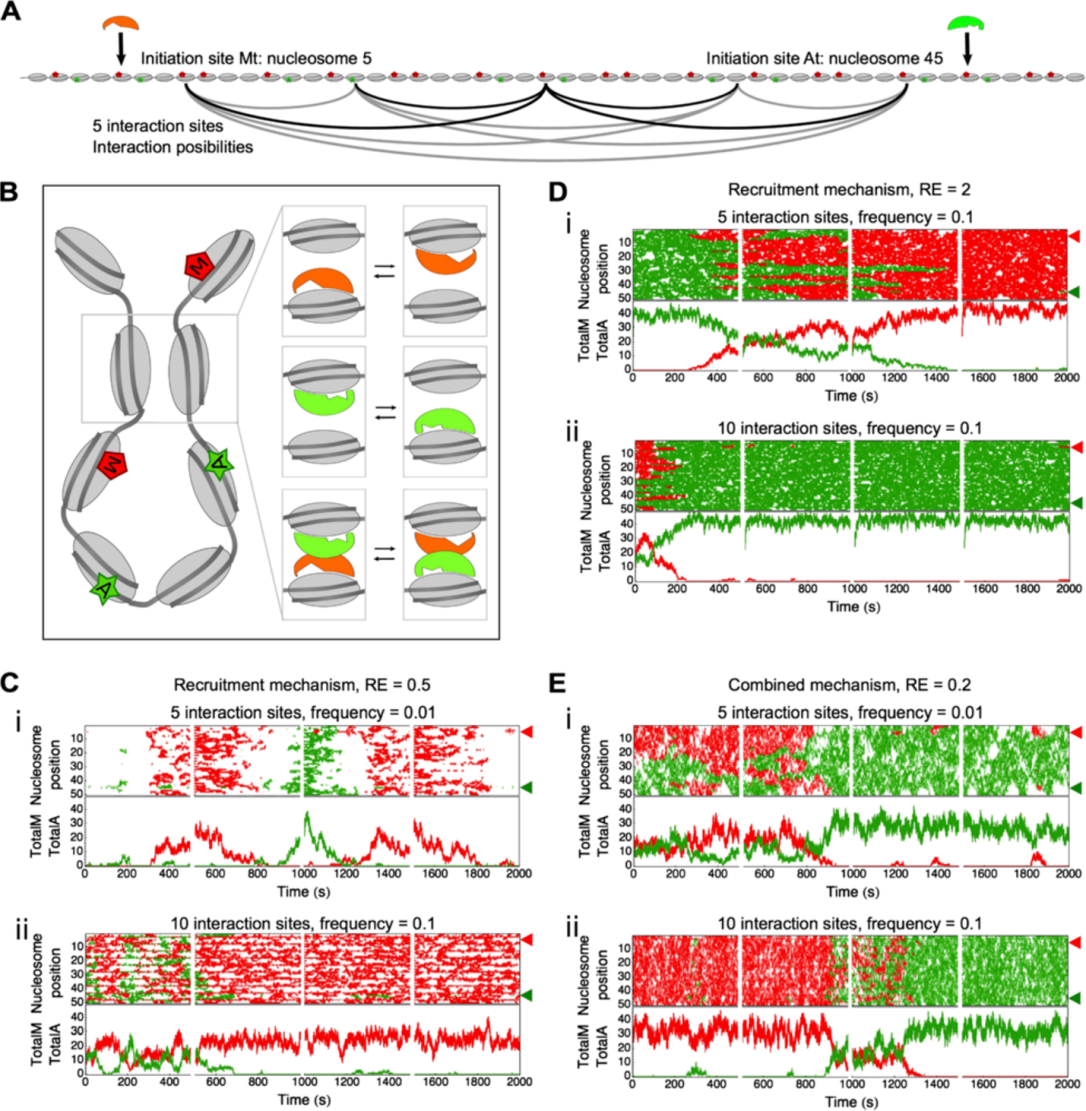
**The emergence of bistability by chromatin connectivity. (A)** The figure represents an example of the nucleosomal array with five interaction sites, in which each interaction site is connected to all other interaction sites. **(B)** The cartoon illustrates that chromatin interactions are initiated when two non-adjacent nucleosomes are connected. If transferases are present on the nucleosomes they are able to hop to the connected nucleosome, or when two different transferases are present they can exchange positions, independent of the nucleosomal modification state (legend see Figure 6). **(C-E)** Each subfigure shows a row of four simulations of 500 s each as an illustration of the model behavior. Top panels show the position of the methylation (red) and acetylation (green) over time, initiation sites indicated by red and green arrowheads (on position 5 and 45, respectively). Bottom panels show the total amount of each modification over time, corresponding to the top panel. The parameters used in these simulations are listed in Table 1. **(C)** Simulation of the recruitment mechanism with recruitment-efficiency 0.5, *k*_*recruitment*_ =1.2 s^-1^. (i) Intermediate connectivity, 10 interaction sites, *k*_*interaction*_ =0.01 s^-1^, (ii) high connectivity, 10 interaction sites, *k*_*interaction*_ =0.1 s^-1^. **(E)** Simulation of the recruitment mechanism with recruitment-efficiency 2, *k*_*recruitment*_ =4.8 s^-1^. (ii) Intermediate connectivity, five interaction sites, *k*_*interaction*_ =0.1 s^-1^, (ii) high connectivity, 10 interaction sites, *k*_*interaction*_ =0.1 s^-1^. **(D)** Simulation of the combined mechanism with recruitment-efficiency 0.2, *k*_*recruitment*_ =0.48 s^-1^. (ii) Intermediate connectivity, five interaction sites, *k*_*interaction*_ =0.01 s^-1^, (ii) high connectivity, 10 interaction sites, *k*_*interaction*_ =0.1 s^-1^. All mechanisms are simulated with two, three, five, and 10 interaction sites equidistant from each other and the border, at *k*_*interaction*_ =0.01 s^-1^ and 0.1 s^-1^. The remainder of the graphs is shown in Additional file 5: Figure S1, Additional file 6: Figure S2, Additional file 7: Figure S3, Additional file 8: Figure S4, and Additional file 9: Figure S5.

In this section, we add chromatin connectivity to the model (Figure 7, Additional file 5: Figure S1, Additional file 6: Figure S2, Additional file 7: Figure S3, Additional file 8: Figure S4, and Additional file 9: Figure S5). We assume that the spatially confined chromatin structure facilitates short distance chromatin-chromatin interactions [51]. We allow reversible chromatin interactions to occur at predefined sites of the nucleosomal array (2, 3, 5, and 10 equidistant sites). In this manner, the connectivity time is exponentially distributed, which can explain experimental connectivity dynamics [51]. We assume that connective sites along the array can initiate chromatin interactions with all other connective sites (Figure 7A). Each pair of interactive sites can form an interaction with each other at rate k_interaction_. When the chromatin interaction is formed, the respective transferase present on the nucleosomes that initiates contact via the interaction either: (i) hops to the other nucleosome it contacts, or (ii) exchanges position with another transferase in case one is present on the other nucleosome (Figure 7B). Although the transferases in the simulations with the recruitment mechanism do not hop or move between nucleosomes, we have chosen to model the connectivity equally for all mechanisms. In the chromatin connectivity simulations the transferases have the same diffusion and recruitment parameters as in the previous section.

Without chromatin connectivity, we hardly find any modifications on the nucleosomal array in the simulations of the opposing transferases with the recruitment mechanism (RE =0.5; Figure 6C). In contrast, upon the introduction of 10 chromatin interaction sites, each with an average interaction frequency of one interaction per 100 s, the system forms highly stochastic modification dynamics with large variability in array-wide methylation and acetylation levels (Figure 7Ci). Around the individual chromatin interaction sites the low activity of recruitment causes dynamic and local methylation and acetylation regions. A higher interaction frequency (10 interaction sites, frequency =0.1 s^-1^) increases the modification extent of the array causing spontaneous state switching between an almost completely methylated and acetylated nucleosomal array (Figure 7Cii).

The introduction of chromatin connectivity in the recruitment mechanism of Figure 6E destabilizes the acetylation-methylation modification boundary. At an intermediate chromatin interaction frequency (at rate 0.1 s^-1^) and with five interaction sites, chromatin connectivity allows acetyl- and methyltransferases to pass over each other and enables the nucleosomal array to switch between alternative states (Figure 7Di). High frequency interactions (10 interaction sites, frequency 0.1 s^-1^) make the state switching more rapid and induce a more stable chromatin composition (Figure 7Dii). In the combined mechanism (Figure 7E) of propagation and low recruitment, the effect of the chromatin connectivity is similar to the results found in Figure 7D. Intermediate connectivity enables faster switching between opposing modifications states and an increased interaction frequency causes switching between nucleosome array states that are almost completely methylated or acetylated (Figure 7Eii). Both in the recruitment mechanism and the combined mechanism, bistability is introduced through chromatin connectivity.

## Discussion

In this study we present a stochastic mathematical model that describes the mechanisms of histone modification spreading. This model incorporates current biological understanding of the processes involved in the spreading of histone modifications, such as diffusion and modification induced recruitment of methyl- and acetyltransferases. We include parameter values that match realistic values enabling the model to describe processes of both dynamic and stable epigenetic pattern formation on both local chromatin sites and larger more widespread chromatin regions.

Previous model efforts have described the dynamics of histone modification state switching. Dodd *et al.* [30] developed a model of the behavior of epigenetic state switching at the mating locus of *Schizosaccharomyces pombe*. In this model of Dodd *et al.*, nucleosomes and their modifications are simplified to three nucleosomal states with state conversions that depend on positive feedback interactions with other nucleosomes in the nucleosomal array. They simplified the model kinetics providing only two possible reactions for a nucleosome: (i) active conversion, driven by positive feedback between two selected nucleosomes, and (ii) noisy conversion of a nucleosome state into a different state. The Dodd model indicates the importance of the feedback-to-noise ratio in the histone modification reactions and the necessity for cooperativity to establish a bistable epigenetic system. Angel *et al.* [35] implemented the Dodd approach to develop a model that explains the *Arabidopsis* epigenetic switch for flowering after an environmental alteration. This *Arabidopsis* model is composed of a two-compartment system in which the nucleation site is modeled as a more interactive region than the surrounding regions. The model allowed the authors to fit the experimentally measured spreading of histone H3 lysine 27 methylation (H3K27me) on the flowering locus. Satake and Iwasa [33] also used a model to simulate *Arabidopsis* during environmental changes and to explain stability of the switch in a stochastic environment. In their study, the rates of modification were formalized as reactions that are dependent on the total amount of modified histones. Thus, in this model a positive feedback-loop was implemented independent of the position of the histones or the mechanism of feedback regulation. In these previous models the molecular interactions of the regulatory proteins (transferases) producing histone modifications were not the object of interest and therefore not mechanically incorporated. Our model extends those studies and considers the kinetic descriptions of nucleosome modifications, that is, diffusive propagation and recruitment of modification enzymes, and chromatin connectivity. As a result, our simulations concern physically realistic parameters, concentrations of modifications enzymes, and time-scales of kinetic and diffusive processes.

All our investigated mechanisms involving transferase propagation (that is, separate or combined diffusion and recruitment as well as chromatin connectivity induced propagation) show that stable patterns of histone modifications can be formed in a dynamic environment. The different mechanisms result in different types of modification patterns. For instance, the diffusion mechanism produces localized patterns around a DNA binding site. These patterns resemble the enrichment of transcription factors commonly found at promoter and enhancer sites of active genes [2, 9]. Additionally, the modification induced recruitment mechanism with a low recruitment-efficiency gives rise to patterns that are similar to patterns generated by the diffusion mechanism. However when the recruitment efficiency is increased, the patterns are widespread and stable. This increased recruitment efficiency corresponds with measured lifetimes of repressive marks that are on average longer than activating histone modifications [52], since long-lived modifications increase the RE of the modification. These widespread patterns are also observed when the mechanisms for diffusion and recruitment are combined together even at low recruitment efficiencies. The latter patterns are reminiscent of histone modifications commonly found at genomic regions consisting of transcriptionally silent genes in which widespread patches of defined histone modification patterns such as H3K9me3 or H3K27me are found [2, 5]. Remarkably, the dynamics of a single transferase in our model is fast, while the stability of the pattern is large. This corresponds with the findings of Cheutin *et al.* [53] showing that even though HP1 exhibits relatively fast dynamic binding kinetics it is involved in creating stable modification patterns.

An important factor for the stability of global modification patterns in the model is the recruitment efficiency. Here, we define this efficiency in terms of two parameters, the lifetime of the modification and the time before recruitment (1/k_demodification_ and 1/k_recruitment_). A change in these parameters can cause large differences in the stability of the modification patterns on the chromatin. Biological alterations in these parameters can be caused by changes in the concentrations of the involved enzymes or changes in their binding affinities. In addition, a change in the binding affinity could be caused by, for example, the presence of another histone modification at the same nucleosome, which could influence the stability of the interaction of the transferase with the nucleosome. Furthermore, we showed that the combined diffusion and recruitment mechanism has a high capability of initiation from a small amount of seeds. These experiments were shown in a more or less neutral background, in which acetylation is not explicitly modeled. This means that although a high seeding capacity is shown, the effects of a heterochromatin protein in an actively acetylated gene could have a different outcome. However, in a favorable environment a fast switch from active to inactive chromatin can be established by the activity of only a few methyltransferases.

Surprisingly, the rate of spreading that we observe in the widespread modification patterns is faster than we expected. In our study, we observe single gene level histone modification pattern formation at a minute scale; around 1 min for the combined mechanism (Figure 3C) and within 5 min in the case of high efficiency recruitment (Figure 4A). Our obtained time scales agree with diffusion times of molecules over chromatin [40, 41]. It should be noted that the mechanics used for this study are simplified in such manner that all protein complex formation and successive binding reactions are not explicitly included. Therefore, the model presented here shows the high end of the possible spreading rates. However, incorporation of complex formation or subsequent binding effects will not cause our model timescale to change from seconds to hours. Hathaway *et al.* [31] developed a model to mechanistically simulate histone modification spreading. They assumed a straightforward neighbor-neighbor interaction of the nucleosomes. With the model assumptions that each nucleosome can modify its neighbor at a certain rate and that each histone can lose its modification at another rate. This model specifically creates a way to study histone modification spreading and pattern formation. The spreading kinetics noticed by Hathaway *et al.* is fitted to their ChIP data of histone modification patterns and provides insight into the average rate of H3K9me3 spreading over the chromatin. The follow-up of this article by Hodges *et al.* [32] showed that the extent of histone modification spreading was restricted intrinsically by the properties of the spreading mechanism. The measurements in the Hathaway [31] article are based on cell population dynamics of histone modifications measured in ChIP experiments, whereas our simulations concern histone modification pattern formation at the level of a single gene. Our study provides insight in histone modification spreading along single genes. The experimental testing of this single gene behavior is an important next step to confirm its biological relevance.

The simulations with two opposing transferases show the capacity of the system to form a natural boundary between an epigenetic active and an inactive stretch of nucleosomes. This is, however, a boundary with an unstable position, indicating that additional control factors are necessary to stabilize the position of the boundary. Two model assumptions are decisive for this (unstable) boundary formation: (i) opposing transferases cannot pass each other on the chromatin causing confinement of the transferases, and (ii) the acetyltransferase and the methyltransferase have the same kinetic properties, which generates a balanced competition of the transferases for the nucleosomes. Interestingly, the system as we show it would be a plausible explanation for early stages in the differential expression of genes that are affected by Position Effect Variegation (PEV). In PEV, genes that are close to an inactive region of the chromatin (mostly telomeric or centromeric) can be differentially expressed in a subpopulation of cells dependent on the dynamic position of the boundary of the heterochromatin region [11, 37].

Similar to computational efforts on histone modification spreading introduced by other authors [30], we find that chromatin connectivity is a mechanism for bistable pattern formation. We show that the introduction of connectivity is necessary for the transferases to pass the boundary and create stretches of fully methylated or fully acetylated chromatin. In nucleosome arrays with two or three interaction sites this process is still quite inefficient but with more interactions, five or 10, and a high interaction-frequency the stability of an active or silenced chromatin state is increased, but also the switch process from one state to the other is faster. During embryonic development gene-expression patterns occur that are cell type specific. Bistability could represent advantageous system behavior, enabling differentiating cells to switch the expression activity of several genes by allowing the chromatin to change its conformation rapidly over a long length scale.

## Conclusion

Our study shows that histone modification patterns can be established within minutes and that nearly irreversible transitions can result, which provides stable epigenetic patterns. Introduction of two opposing enzymes causes dynamic histone modification boundaries whereas chromatin connectivity can introduce both bistable epigenetic state switching and stable histone modification patterns. We demonstrate in our model that with biologically plausible parameter regimes both epigenetic stochastic switching and stable pattern formation are noticed to provide cell-to-cell heterogeneity and robustness in gene expression.

## Methods

### Model parameters

The reaction parameters in the model are derived from physical and biological constants. We have taken some assumptions to derive these parameters. The association and background modification reactions in the model are based on diffusion limited association reactions. The reaction rate constant is dependent on the concentration of the enzyme, the diffusion constants of the DNA and the enzyme, the diameter of the binding site and the enzyme, and on the volume of the nucleus.


Here  is the apparent first-order association rate constant for the binding of modification enzymes to a DNA site, k_on_ is the second-order diffusion limited rate constant, n_E_ is the number of modification enzymes per nucleus with volume V_nucleus_, D_E_ and D_S_ are the diffusion coefficients of the enzyme and the DNA site, and r_E_ and r_S_ are the radii of the enzyme and the DNA site. Since we fix the total number of modification enzymes the relevant rate constant to consider is .

The diffusion constant of enzymes (D_E_) in a cell are between 0.5 and 5 μm^2^s^-1^ [54]. The diffusion constant of the DNA binding site (*D*_*S*_) is expected to be a few orders of magnitude lower than the diffusion constant of the enzyme, therefore we neglect this constant. We assume a transferase of about 5 nm in diameter and a DNA binding site of 15 bp, which measures 5 nm. The volume of an average human nucleus is 500 pL. The concentration of specific histone modification transferases in the nucleus has not been reported so far. Transcription factors are estimated to be present in a wide copy range from tens to thousands per cell [23]. Here we assume that 5,000 copies of modification and demodification enzymes are present per cell. Together these values give an apparent diffusion limited association rate of 2.4 s^-1^. This value is used for the binding at the initiation site, the rate of demodification and the rate of modification for the acetyltransferase in the first section of the results (parameters k_on_, k_demodification_, and k_modification_).

The transferase nucleosomal release rate is based on FRAP analysis of transcription factors and histone modification transferases [55]. The residence time of different factors ranges from milliseconds to several minutes. HP1β is shown to have a chromatin binding residence time of 11.3 s in the fast fraction, covering 88% of the HP1β population. We consider that the transferase has an average residence time of 10 s on the nucleosome array. Thus, the parameter for the transferase detaching from the nucleosome (k_off_) is 0.1 s^-1^.

Gorman *et al*. [40, 41] studied 1D sliding of proteins on the DNA. Several different proteins have been studied in an *in vitro* setting and their diffusion coefficients have been measured. Measured diffusion coefficients range from 2 × 10^-4^ μm^2^s^-1^ to 0.5 μm^2^s^-1^. For our model we assume the lower end of the range because most of the measurements were performed on stretched, nucleosome free DNA while the measurements on chromatin gave varying results, depending on the measured transferase [41]. The diffusion parameter is defined as the rate of movement over the approximately 25 nm distance from nucleosome to nucleosome, resulting in a diffusion parameter (k_slide_) of 0.6 s^-1^.

We assume that the transferase modification reaction is fast once the transferase is bound to the nucleosome (k_transferase_ =1,000 s^-1^). The transferase neighbor modification reactions however are much slower due to the persistence length of the DNA over short distances (k_neighbor_ =0.2 s^-1^). From these assumptions we calculated the model parameters (Table 1). Although we made assumptions in the parameter set, our model is able to describe the dynamics of the system based on biological data in a qualitative manner. A description for the NucleosomeModelBuilder, that is part of the NucleosomeTool, is included in Additional file 1: S1. The reactions used to describe the model are summarized in Additional file 10: S5.

### StochPy data analysis and probability distributions

The model with different mechanisms and parameter settings was simulated with the NucleosomeTool plug-in designed for StochPy [43]. StochPy uses the Gillespie algorithm for the stochastic simulations. We selected the next reaction method for all simulations in this study. StochPy and the NucleosomeTool are available from: http://stochpy.sourceforge.net/. Simulation data are saved and imported into *Mathematica* (Wolfram Research) for further analysis.

Simulations with one transferase are run for at least 1,000 time trajectories of at least 400 s simulated time. This accounts for 34 to 89 million data points per simulated mechanism. Dynamics plots (Figure 3 column ii and iv) are calculated by binning of data into 1 s bins and then calculation of lowest value, highest value, median, and 0.25 and 0.75 quartile range of the data points per bin. These are plotted as error bars to the median value.

To create the plots of steady-state probabilities of the model species the simulations are repeated 1,000 times with an initial state taken from an average steady-state situation. Pattern distributions (Figure 3 column i) are calculated from the final 60% of the steady-state data to ensure steady state are reached. The nucleosomes in each state are multiplied by the time spent in this state divided by the total time to calculate the probability of each state at each nucleosome position. Probability distribution (Figure 3 column iii) of the steady-state simulations is obtained by binning the total number of nucleosomes in each state (M, A, or U) and calculating the time spent in each total amount divided by the total time. Of the steady-state simulations only the final 60% of the simulated data are used to ensure steady-state values are calculated.

In the simulations with two opposing enzymes, lowering the initiation parameter from 2.4 s^-1^ to 0.01 s^-1^ created the opportunity for one transferase to completely occupy the array. Because the computational limits of our system are reached at approximately 600 to 800 model seconds the simulations are run for 500 s. Figures 6 and 7 contain simulations of 500 s that are placed adjacently to give an illustration of long-term model behavior.

## Authors’ information

PJV (Associate Professor and Group leader) investigates functional and dynamic organization of the mammalian genome exploring the use of engineering (synthetic) mammalian cell systems allowing to modulate the epigenetic state performing quantitative single-cell measurements and computational stochastic modeling. Her main research focus is on epigenetic state switching and transcriptional dynamics/stochasticity also in the context of deregulated behavior such as UV-induced DNA damage repair and age-related diseases.

FJB (Associate and Extraordinary Professor) uses theory and experiments to study the mechanisms and principles of cellular adaptation to new conditions developing simulation software for combined simulation of stochasticity of molecular reactions and cell growth. Main research topics include metabolic regulation by gene expression, mixing of transcription stochasticity and cell-growth stochasticity, and genome-wide adaptation of metabolism.

LCMA and TRM are PhD students using systems biology approaches to understand complex biological behavior.

## Electronic supplementary material

Additional file 1: S1: Description of NucleosomeTool plug-in. (DOCX 36 KB)

Additional file 2: S2: Scripts for NucleosomeTool usage. (DOCX 18 KB)

Additional file 3: S3: Scripts S1 Python scripts to regenerate Figures 1, 2, 3, 4, 5, 6, and 7 from the used stochastic models in the NucleosomeTool. Further details can be found in Additional file 1: S1 and Additional file 2: S2. (ZIP 1 KB)

Additional file 4: S4: Scripts S2 Python scripts to regenerate Additional file 5: Figure S1, Additional file 6: Figure S2, Additional file 7: Figure S3, Additional file 8: Figure S4 and Additional file 9: Figure S5 from the used stochastic models in the NucleosomeTool. Further details can be found in Additional file 1: S1 and Additional file 2: S2. (ZIP 1 KB)

Additional file 5: Figure S1: The influence of chromatin connectivity on the diffusion mechanism, related to Figure 7. (AII) The figure shows simulation of the diffusion mechanism. Each subfigure shows a row of four simulations of 500 s each as an illustration of the model behavior. Top panels of each subfigure show the position (y-axis) of the methylation (red) and acetylation (green) over time (x-axis), initiation sites indicated by red and green arrowheads (on positions 5 and 45, respectively). Bottom panels show the total amount of each modification over time, corresponding to the top panel. Left column figures (B, D, F, H) show interaction at frequency *k*_*interaction*_ =0.01 s^-1^, right column figures (C, E, G, I) show interaction at frequency *k*_*interaction*_ =0.1 s^-1^. The other parameters used in these simulations are listed in Table 1. (A) Zero interaction sites. (B, C) Two interaction sites at positions 15 and 35. (D, E) Three interaction sites at positions 12, 25, and 38. (F, G) Five interaction sites at positions 8, 16, 25, 34, and 42. (H, I) Ten interaction sites at positions 3, 8, 13, 18, 23, 28, 33, 38, 43, and 48. (JPEG 679 KB)

Additional file 6: Figure S2: The influence of chromatin connectivity on the recruitment mechanism (RE =0.5), related to Figure 7. (A-I) The figure shows simulation of the modification induced recruitment mechanism with recruitment-efficiency 0.5 (k_recruitment_ =1.2 s^-1^). Each subfigure shows a row of four simulations of 500 s each as an illustration of the model behavior. Top panels of each subfigure show the position (y-axis) of the methylation (red) and acetylation (green) over time (x-axis), initiation sites indicated by red and green arrowheads (on positions 5 and 45, respectively). Bottom panels show the total amount of each modification over time, corresponding to the top panel. Left column figures (B, D, F, H) show interaction at *k*_*interaction*_ =0.01 s^-1^, right column figures (C, E, G, I) show interaction at *k*_*interaction*_ =0.1 s^-1^. The other parameters used in these simulations are listed in Table 1. (A) Zero interaction sites. (B, C) Two interaction sites at positions 15 and 35. (D, E) Three interaction sites at positions 12, 25, and 38. (F, G) Five interaction sites at positions 8, 16, 25, 34, and 42. (H, I) Ten interaction sites at positions 3, 8, 13, 18, 23, 28, 33, 38, 43, and 48. (JPEG 1 MB)

Additional file 7: Figure S3: The influence of chromatin connectivity on the recruitment mechanism (RE =2), related to Figure 7. (A-I) The figure shows simulation of the modification induced recruitment mechanism with recruitment-efficiency 2 (k_recruitment_ =4.8 s^-1^). Each subfigure shows a row of four simulations of 500 s each as an illustration of the model behavior. Top panels of each subfigure show the position (y-axis) of the methylation (red) and acetylation (green) over time (x-axis), initiation sites indicated by red and green arrowheads (on positions 5 and 45, respectively). Bottom panels show the total amount of each modification over time, corresponding to the top panel. Left column figures (B, D, F, H) show interaction at *k*_*interaction*_ =0.01 s^-1^, right column figures (C, E, G, I) show interaction at *k*_*interaction*_ =0.1 s^-1^. The other parameters used in these simulations are listed in Table 1. (A) Zero interaction sites. (B, C) Two interaction sites at positions 15 and 35. (D, E) Three interaction sites at positions 12, 25, and 38. (F, G) Five interaction sites at positions 8, 16, 25, 34, and 42. (H, I) Ten interaction sites at positions 3, 8, 13, 18, 23, 28, 33, 38, 43, and 48. (JPEG 1 MB)

Additional file 8: Figure S4: The influence of chromatin connectivity on the combined mechanism (RE =0.1), related to Figure 7. (A-I) The figure shows simulation of the combined mechanism with recruitment-efficiency 0.1 (k_recruitment_ =0.24 s^-1^). Each subfigure shows a row of four simulations of 500 s each as an illustration of the model behavior. Top panels of each subfigure show the position (y-axis) of the methylation (red) and acetylation (green) over time (x-axis), initiation sites indicated by red and green arrowheads (on positions 5 and 45, respectively). Bottom panels show the total amount of each modification over time, corresponding to the top panel. Left column figures (B, D, F, H) show interaction at *k*_*interaction*_ =0.01 s^-1^, right column figures (C, E, G, I) show interaction at *k*_*interaction*_ =0.1 s^-1^. The other parameters used in these simulations are listed in Table 1. (A) Zero interaction sites. (B, C) Two interaction sites at positions 15 and 35. (D, E) Three interaction sites at positions 12, 25, and 38. (F, G) Five interaction sites at positions 8, 16, 25, 34, and 42. (H, I) Ten interaction sites at positions 3, 8, 13, 18, 23, 28, 33, 38, 43, and 48. (JPEG 882 KB)

Additional file 9: Figure S5: The influence of chromatin connectivity on the combined mechanism (RE =0.2), related to Figure 7. (A-I) The figure shows simulation of the combined mechanism with recruitment-efficiency 0.2 (k_recruitment_ =0.48 s^-1^). Each subfigure shows a row of four simulations of 500 s each as an illustration of the model behavior. Top panels of each subfigure show the position (y-axis) of the methylation (red) and acetylation (green) over time (x-axis), initiation sites indicated by red and green arrowheads (on positions 5 and 45, respectively). Bottom panels show the total amount of each modification over time, corresponding to the top panel. Left column figures (B, D, F, H) show interaction at *k*_*interaction*_ =0.01 s^-1^, right column figures (C, E, G, I) show interactions at *k*_*interaction*_ =0.1 s^-1^. The other parameters used in these simulations are listed in Table 1. (A) Zero interaction sites. (B, C) Two interaction sites at positions 15 and 35. (D, E) Three interaction sites at positions 12, 25, and 38. (F, G) Five interaction sites at positions 8, 16, 25, 34, and 42. (H, I) Ten interaction sites at positions 3, 8, 13, 18, 23, 28, 33, 38, 43, and 48. (JPEG 2 MB)

Additional file 10: S5: Summarized list of model reactions. (DOCX 19 KB)

## Abbreviations

A: Acetylated or active nucleosome state
At: Acetyltransferase
ChIP: Chromatin ImmunoPrecipitation
ChIP-sec: Chromatin ImmunoPrecipitation followed by sequencing of the obtained DNA fragments
H3K27me: Histone H3 lysine 27 methylation
H3K9me3: Histone H3 trimethylated at lysine 9 HP1, Heterochromatin Protein 1
M: Methylated or silenced nucleosome state
Mt: Methyltransferase
NFR: Nucleosome-free region
PEV: Position effect variegation
RE: Recruitment efficiency
Suv(3-9)h1: The mammalian ortholog of *Drosophila* suppressor of variegation 3-9 (Su(var)3-9)
U: Unmodified or neutral nucleosome state.
