# Supplementary material for: Mechanistic stochastic model of histone modification pattern formation
Source: Epigenetics Chromatin. 2014 Oct 27;7:30. doi: 10.1186/1756-8935-7-30 (PMC4234852; doi:10.1186/1756-8935-7-30)
Supplement: Supplementary file 1 — Additional file 1: S1: Description of NucleosomeTool plug-in. (DOCX 36 KB) [file 13072_2014_336_MOESM1_ESM.docx]

**Additional Information**

**S1. Description and usage of NucleosomeTool plug-in.**

In this study, we used the StochPy (Stochastic modeling in Python) [1] software package for stochastic simulation. For our specific purposes, we have developed a NucleosomeTool plug-in for StochPy, which has modules for both building multi-nucleosome models and simulation of those models. Both our plug-in and StochPy are available for download from http://stochpy.sf.net).

*NucleosomeModelBuilder* is the module within the NucleosomeTool plug-in that constructs models in the human-readable/writeable PySCeS model description language (MDL) [2]. Each model consists of reactions, parameters, and initial conditions with the given features. Possible features are: model name, number of nucleosomes, mode of transferase movement (1D diffusion and/or recruitment), explicit tracking of methyltransferase Mt, acetyltransferase At or both, looping sites, and initial conditions for the model. Box S1 gives an example of the usage of the *NucleosomeModelBuilder*. By default, the *NucleosomeModelBuilder* creates the model used to generate the results shown in Figure 2 of the main text.

**Feature description**

**Box S1. Example of Nucleosome Builder usage:**

import stochpy
bmod=stochpy.NucleosomeModelBuilder()

bmod.SetNucleosomes(30)
bmod.SetInitiationSites({'M':[5],'A':[26]})
bmod.SetSliding(True)
bmod.SetRecruitment(False) bmod.SetLoopSites([10,20])
bmod.SetInitialState('A')
bmod.BuildModel(File='model_new')

Use the first two lines of the script in Box S1 to import StochPy and to start the *NucleosomeModelBuilder*

*SetNucleosome(n)*: changes the number of nucleosomes in the model. Default is 50 nucleosomes.

*SetInitiationSites(dict):* specifies the transferases to track explicitly and the nucleosomes used as initiation site(s) in the form of a Python dictionary. The default is *dict* = {'M':[25]}; methyltransferases at initiation site 25. The model created in Box S1 will have both a methyl- and acetyltransferase with initiation sites at nucleosomes 5 and 26, respectively. Note that more than one initiation site can be given per transferase. For example, {'M':[2,4,6], 'A':[1,3,4]} will create a model in which initiation sites for Mt are 2, 4, and 6 and for At are 1, 3, and 4.

*SetSliding()* and *SetRecruitment()*: toggles the movement properties of the transferases where True is on and False is off. By default, sliding is on and recruitment is off, at least one should be included for transferase movement. *IsSliding(True)* will generate diffusion reactions for all specified transferases. Alternatively, *IsRecruitment(True)* will generate both the recruitment reactions and the transferases ability to modify its neighbor.

*SetLoopSites(list):* specifies the interaction sites in the model. If you include any interaction sites you have to specify at least two locations in a Python list. If the string ‘all’ is given the builder will automatically loop all nucleosomes to all others (beware this feature adds a huge amount of reactions to the model which might cause computational problems). The default setting is no interactions and the example given in Box S1 will create loop sites 10 and 20, these will now interact with each other.

*SetInitialState(state):* the initial conditions for the array of nucleosomes. The default setting is 'U', all nucleosomes in unmodified state. Input of this function is specified as a string, all nucleosomes will start as: 'M', 'MtM', 'A', 'AtA', 'U', or 'random'. In the example given in Box S1, all nucleosomes will start as A without any transferase.

The parameters have, by default, the following values: Kon = 2.4, Koff = 0.1, Kmodification = 2.4, Kdemodification= 2.4, Kslide = 0.6, Ktransferase = 1000.0, Krecruitment = 1.2, Kneighbor = 0.2, Kloop = 0.01. Parameter values can be modified: (1) when one starts the *NucleosomeModelBuilder*, or (2) interactively by using the high-level function *ChangeParameter*. Alternatively, the parameter values can easily be changed in the model file afterwards.

After specifying the model, *BuildModel* creates the model and stores it in the StochPy model directory. If no argument is provided, the model will be called ‘model_test.psc’.

**NucleosomeSimulator**

The *NucleosomeSimulator* is specifically developed to simulate and analyze the models generated by the *NucleosomeModelBuilder*. This simulator is hard wired to: (1) handle the three states of the nucleosomes and two kinds of transferases, and (2) create time-dependent plots and distribution plots. Two types of stochastic simulations are possible in this module: (1) mesoscopic simulations that, in addition to saving the total M, A, U, Mt, and At at each time point, will store states of all nucleosomes at all time points, and (2) macroscopic simulations that only saves the total M, A, U, Mt, and At at each time point. This macroscopic simulation tool will allow for longer simulations, whereas with less detailed output.

Upon installation of the NucleosomeTool a model file is created that contains the model that was used to create Figure 2 and Figure 3A of the main text. This model will be loaded by default by the *NucleosomeSimulator*.

**Feature description**

*Model(File,dir*): imports the specified model file into StochPy.

**Box S2. Example of NucleosomeSimulator usage**:

import stochpy
smod=stochpy.NucleosomeSimulator()

smod.DoMesoscopicStochSim(mode='time',end=100)

smod.PlotPatternTimeSeries()
smod.PlotSpeciesTimeSeries()
smod.PlotSpeciesDistributions()

*ChangeParameter(parameter,value):* allows for interactive change of model parameters. Both the parameter name and the new value must be provided.

*DoMesoscopicStochSim():* after this command StochPy will run a stochastic simulation for the default or user-specified settings. This high-level function will take arguments to specify the end of simulation (end = integer) and the mode of keeping time (mode = 'time' or 'steps'); the example of Box S2 will run a simulation until t = 100. In this simulation, all states for all nucleosomes are saved, for example, M1, M2, …, U1, U2, …, A1, A2, …, A50. Users are advised to initially test a short simulation time (for example, time = 50 or steps = 1,000) to estimate computation times of longer simulations.

*DoMacroscopicStochSim()*: this high-level function works in a similar fashion as *DoMesoscopicStochSim,* except that only the total M, U, A, Mt, and At are stored. Note that, therefore, note all plotting options are available. Again, users are advised to initially test a short simulation time (for example, time = 50 or steps = 1,000) to estimate computation times of longer simulations.

Also, several plotting options are available in the *NucleosomeSimulator*:

*PlotPatternTimeSeries()*: creates a figure comparable to Figure 2 where for each time point in the time series (x-axis) the modification state at each position (y-axis) is plotted in red for M and green for A. Colors of the plot may be changed (for example, Mcolor = 'blue', Acolor = 'yellow').

*PlotPatternDistributions()*: creates a plot resembling to Figure 3 column i. It shows the pattern created on the linear array for each species calculated over the total simulation time.

*PlotSpeciesTimeSeries()*: creates a plot similar to that of Figure 3 column ii. It shows the total amount of each species over time.

*PlotSpeciesDistributions():* Plots the distribution of each species calculated over the simulated time. Beware that the initial conditions could cause the distribution to be somewhat skewed if not enough time steps are simulated.

Alternatively, the functions *ExportMeso2File()* and *ExportMacro2File()* allow users to export their simulations to a text file.

**Usage**

Using the following script, Figure 2 can be recreated:

import stochpy
smod=stochpy.NucleosomeSimulator(File='model_test.psc')
smod.DoMesoscopicStochSim(mode='time',end=100)
smod.PlotPatternTimeSeries()

Three extra plot options create Figure 3Ai, Figure 3Aii, and Figure 3Aiii

smod.PlotPatternDistributions()
smod.PlotSpeciesTimeSeries()
smod.PlotSpeciesDistributions()

Figure 3Aiv can be recreated by creating a new model file:

bmod=stochpy.NucleosomeModelBuilder()
bmod.SetInitialState('MtM')

bmod.BuildModel('model_Fig3Aiv')

And loading this new model into the NucleosomeSimulator

smod.Model('model_Fig3Aiv.psc')

smod.DoMesoscopicStochSim(mode='time',end=100)
smod.PlotSpeciesTimeSeries()

Scripts S1 contains scripts to regenerate all models used in the article.

**References**

1. Maarleveld TR, Olivier BG, Bruggeman FJ: **StochPy: A Comprehensive, User-Friendly Tool for Simulating Stochastic Biological Processes.** *PLoS One* 2013, **8:**e79345.

2. Olivier BG, Rohwer JM, Hofmeyr J-HS: **Modelling cellular systems with PySCeS.** *Bioinformatics* 2005, **21:**560–561.
