# Supplementary material for: Mechanistic stochastic model of histone modification pattern formation
Source: Epigenetics Chromatin. 2014 Oct 27;7:30. doi: 10.1186/1756-8935-7-30 (PMC4234852; doi:10.1186/1756-8935-7-30)
Supplement: Supplementary file 2 — Additional file 2: S2: Scripts for NucleosomeTool usage. (DOCX 18 KB) [file 13072_2014_336_MOESM2_ESM.docx]

**Additional Information S2.**

**Scripts for building the models used in the article**

The Python scripts provided in Scripts S1 (NTbuild.py and NTbuild_supp.py) contain the scripts for building all models used in this article. NTbuild.py will build all models for the figures in the main text. NTbuild_supp.py builds the additional models shown in the Supporting Figures. The .psc output files are all named in the following format: 'model_Fig*i*.psc' where *i* is the subfigure number, for example, the model for Figure 3Ai to 3Aiii is saved as 'model_Fig3.psc' and Figure 5Dii is saved as 'model_Fig5Dii.psc'.

**Example of usage**

The NTbuild.py file can be run in Python by the following command:

Python NTbuild.py

All .psc files are now saved in the StochPy/pscmodels folder. Using the following script in Python, Figure 3Bi-3Biii can be recreated:

import stochpy
smod=stochpy.NucleosomeSimulator(File='model_Fig3B.psc')
smod.DoMesoscopicStochSim(mode='time',end=100)
smod.PlotPatternDistributions()
smod.PlotSpeciesTimeSeries()
smod.PlotSpeciesDistribution()

Figure 3Biv must be loaded separately into NucleosomeSimulator:

smod.Model('model_Fig3Biv.psc')
smod.DoMesoscopicStochSim(mode='time',end=100)
smod.PlotSpeciesTimeSeries()
