# Supplementary figures and images for: Mechanistic stochastic model of histone modification pattern formation
Source: Epigenetics Chromatin. 2014 Oct 27;7:30. doi: 10.1186/1756-8935-7-30 (PMC4234852; doi:10.1186/1756-8935-7-30)

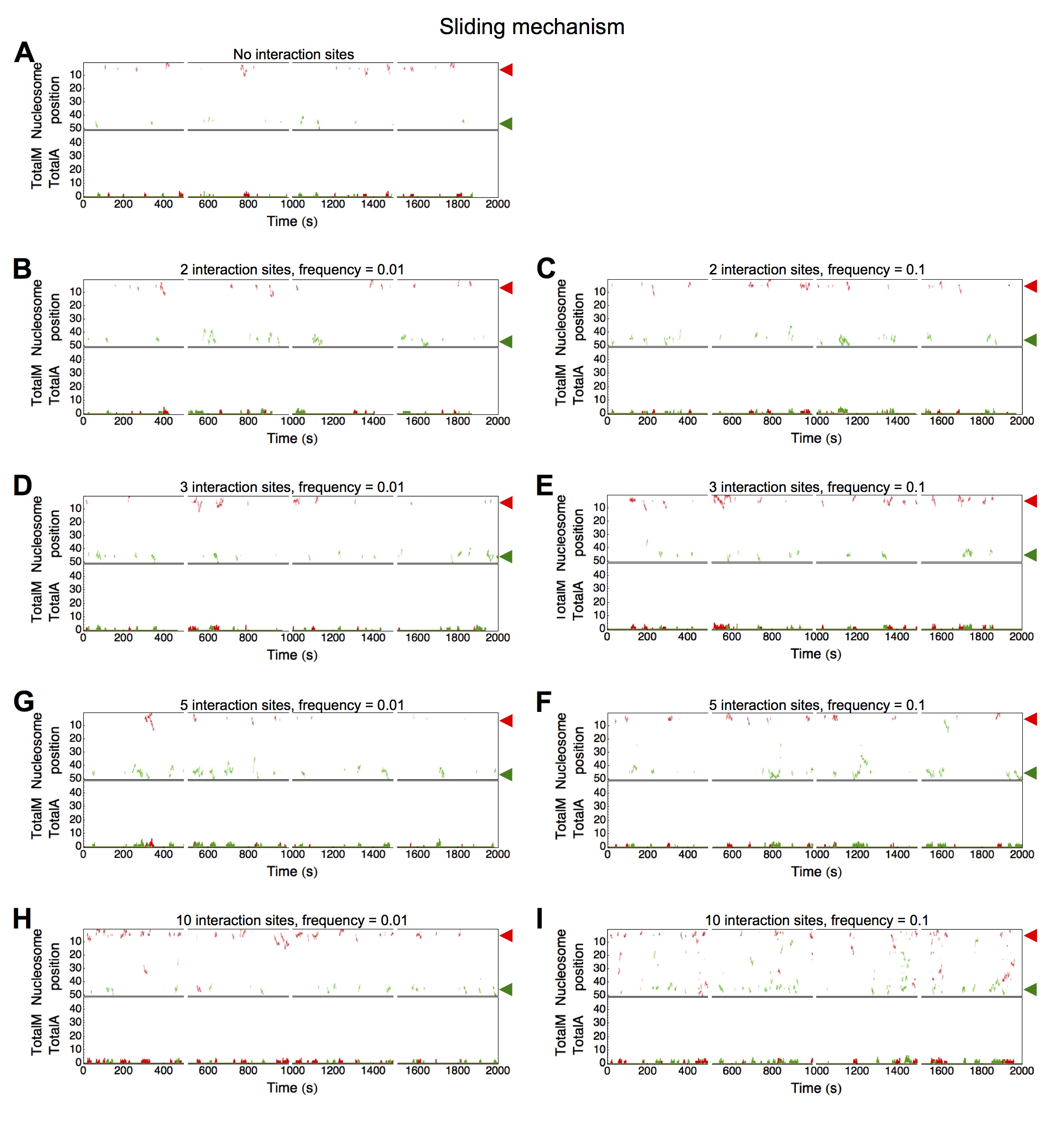

Supplement: Supplementary file 5 — Additional file 5: Figure S1: The influence of chromatin connectivity on the diffusion mechanism, related to Figure 7. (AII) The figure shows simulation of the diffusion mechanism. Each subfigure shows a row of four simulations of 500 s each as an illustration of the model behavior. Top panels of each subfigure show the position (y-axis) of the methylation (red) and acetylation (green) over time (x-axis), initiation sites indicated by red and green arrowheads (on positions 5 and 45, respectively). Bottom panels show the total amount of each modification over time, corresponding to the top panel. Left column figures (B, D, F, H) show interaction at frequency k interaction =0.01 s-1, right column figures (C, E, G, I) show interaction at frequency k interaction =0.1 s-1. The other parameters used in these simulations are listed in Table 1. (A) Zero interaction sites. (B, C) Two interaction sites at positions 15 and 35. (D, E) Three interaction sites at positions 12, 25, and 38. (F, G) Five interaction sites at positions 8, 16, 25, 34, and 42. (H, I) Ten interaction sites at positions 3, 8, 13, 18, 23, 28, 33, 38, 43, and 48. (JPEG 679 KB) [file 13072_2014_336_MOESM5_ESM.jpeg]

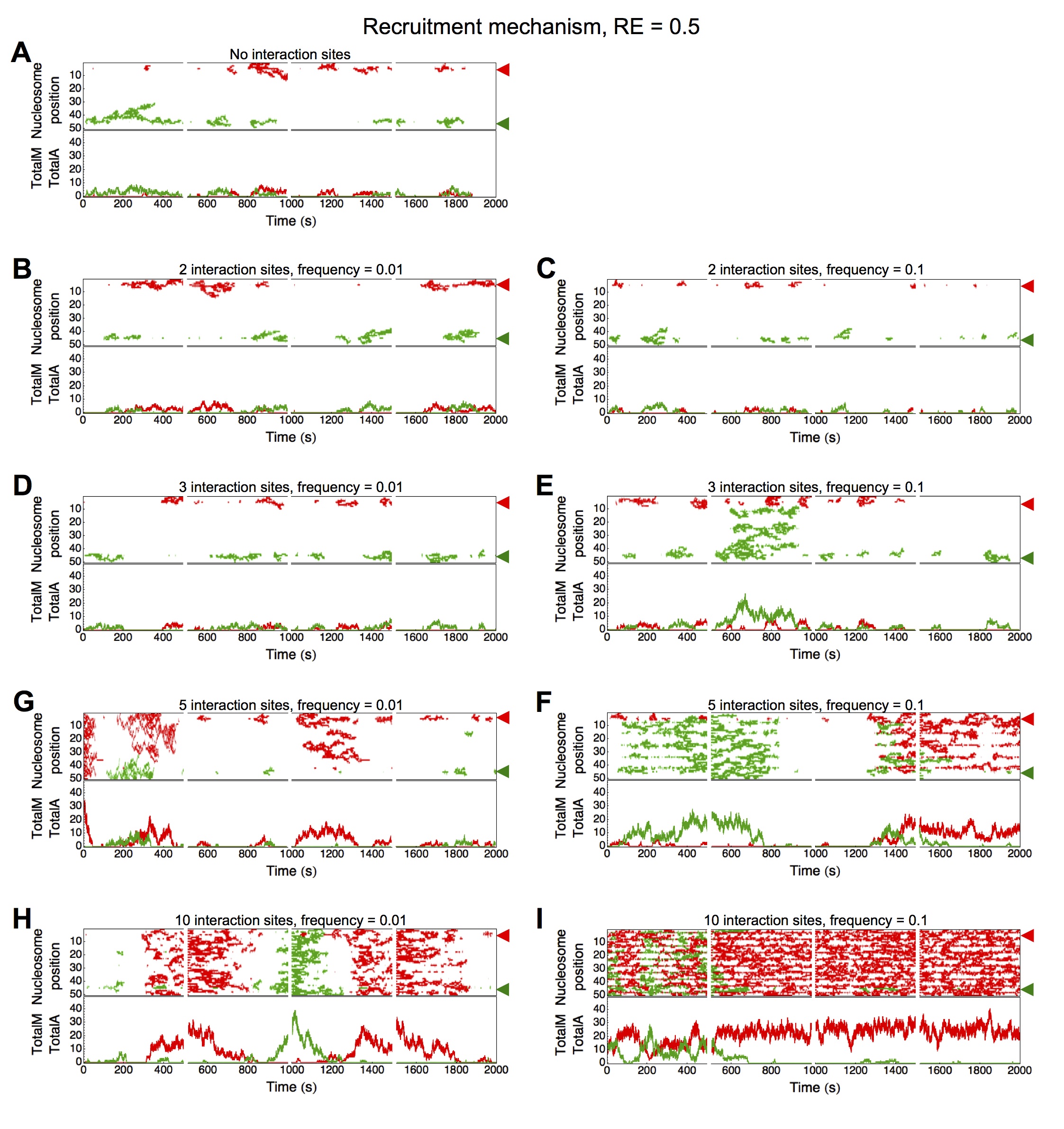

Supplement: Supplementary file 6 — Additional file 6: Figure S2: The influence of chromatin connectivity on the recruitment mechanism (RE =0.5), related to Figure 7. (A-I) The figure shows simulation of the modification induced recruitment mechanism with recruitment-efficiency 0.5 (krecruitment =1.2 s-1). Each subfigure shows a row of four simulations of 500 s each as an illustration of the model behavior. Top panels of each subfigure show the position (y-axis) of the methylation (red) and acetylation (green) over time (x-axis), initiation sites indicated by red and green arrowheads (on positions 5 and 45, respectively). Bottom panels show the total amount of each modification over time, corresponding to the top panel. Left column figures (B, D, F, H) show interaction at k interaction =0.01 s-1, right column figures (C, E, G, I) show interaction at k interaction =0.1 s-1. The other parameters used in these simulations are listed in Table 1. (A) Zero interaction sites. (B, C) Two interaction sites at positions 15 and 35. (D, E) Three interaction sites at positions 12, 25, and 38. (F, G) Five interaction sites at positions 8, 16, 25, 34, and 42. (H, I) Ten interaction sites at positions 3, 8, 13, 18, 23, 28, 33, 38, 43, and 48. (JPEG 1 MB) [file 13072_2014_336_MOESM6_ESM.jpeg]

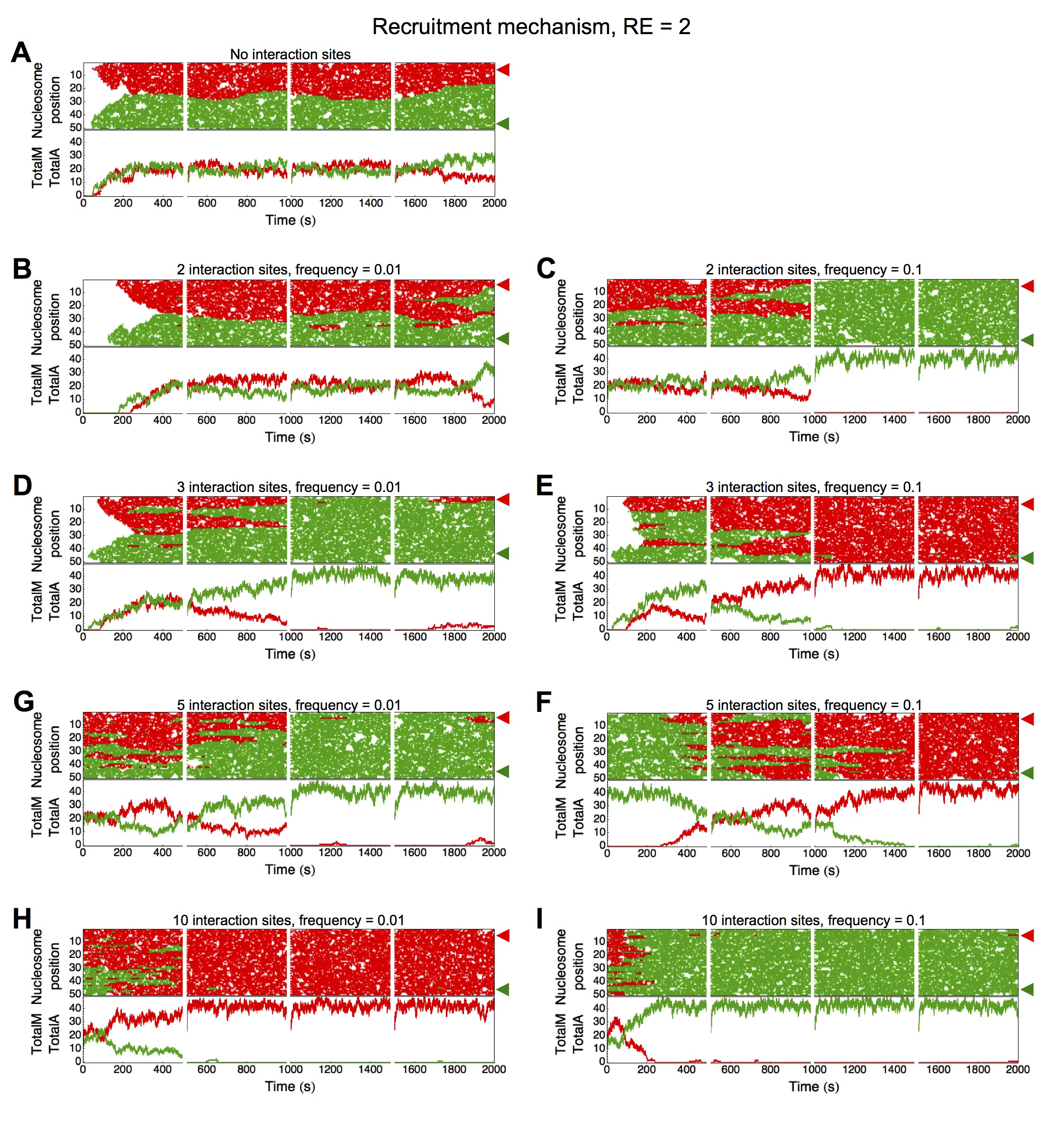

Supplement: Supplementary file 7 — Additional file 7: Figure S3: The influence of chromatin connectivity on the recruitment mechanism (RE =2), related to Figure 7. (A-I) The figure shows simulation of the modification induced recruitment mechanism with recruitment-efficiency 2 (krecruitment =4.8 s-1). Each subfigure shows a row of four simulations of 500 s each as an illustration of the model behavior. Top panels of each subfigure show the position (y-axis) of the methylation (red) and acetylation (green) over time (x-axis), initiation sites indicated by red and green arrowheads (on positions 5 and 45, respectively). Bottom panels show the total amount of each modification over time, corresponding to the top panel. Left column figures (B, D, F, H) show interaction at k interaction =0.01 s-1, right column figures (C, E, G, I) show interaction at k interaction =0.1 s-1. The other parameters used in these simulations are listed in Table 1. (A) Zero interaction sites. (B, C) Two interaction sites at positions 15 and 35. (D, E) Three interaction sites at positions 12, 25, and 38. (F, G) Five interaction sites at positions 8, 16, 25, 34, and 42. (H, I) Ten interaction sites at positions 3, 8, 13, 18, 23, 28, 33, 38, 43, and 48. (JPEG 1 MB) [file 13072_2014_336_MOESM7_ESM.jpeg]

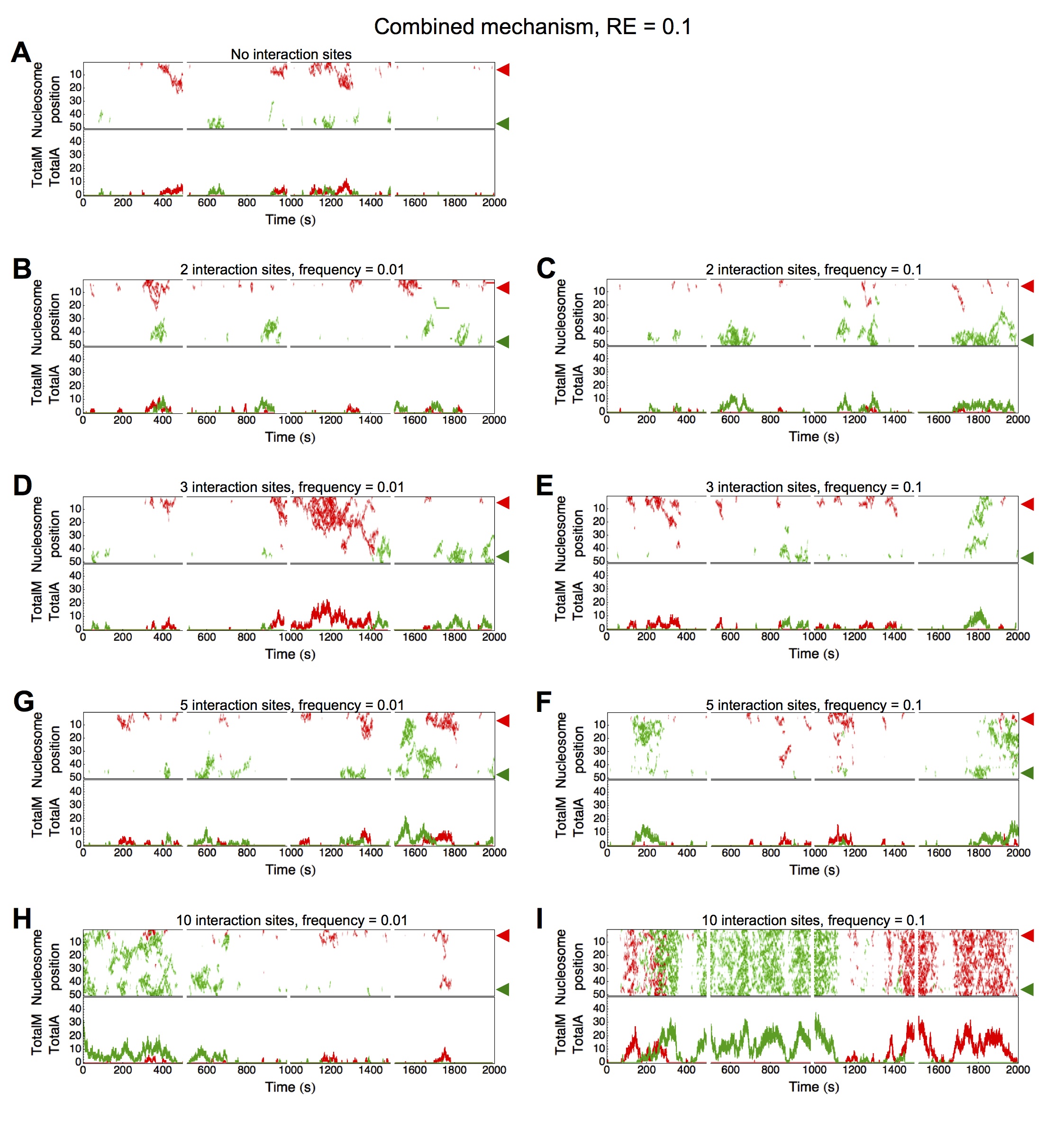

Supplement: Supplementary file 8 — Additional file 8: Figure S4: The influence of chromatin connectivity on the combined mechanism (RE =0.1), related to Figure 7. (A-I) The figure shows simulation of the combined mechanism with recruitment-efficiency 0.1 (krecruitment =0.24 s-1). Each subfigure shows a row of four simulations of 500 s each as an illustration of the model behavior. Top panels of each subfigure show the position (y-axis) of the methylation (red) and acetylation (green) over time (x-axis), initiation sites indicated by red and green arrowheads (on positions 5 and 45, respectively). Bottom panels show the total amount of each modification over time, corresponding to the top panel. Left column figures (B, D, F, H) show interaction at k interaction =0.01 s-1, right column figures (C, E, G, I) show interaction at k interaction =0.1 s-1. The other parameters used in these simulations are listed in Table 1. (A) Zero interaction sites. (B, C) Two interaction sites at positions 15 and 35. (D, E) Three interaction sites at positions 12, 25, and 38. (F, G) Five interaction sites at positions 8, 16, 25, 34, and 42. (H, I) Ten interaction sites at positions 3, 8, 13, 18, 23, 28, 33, 38, 43, and 48. (JPEG 882 KB) [file 13072_2014_336_MOESM8_ESM.jpeg]

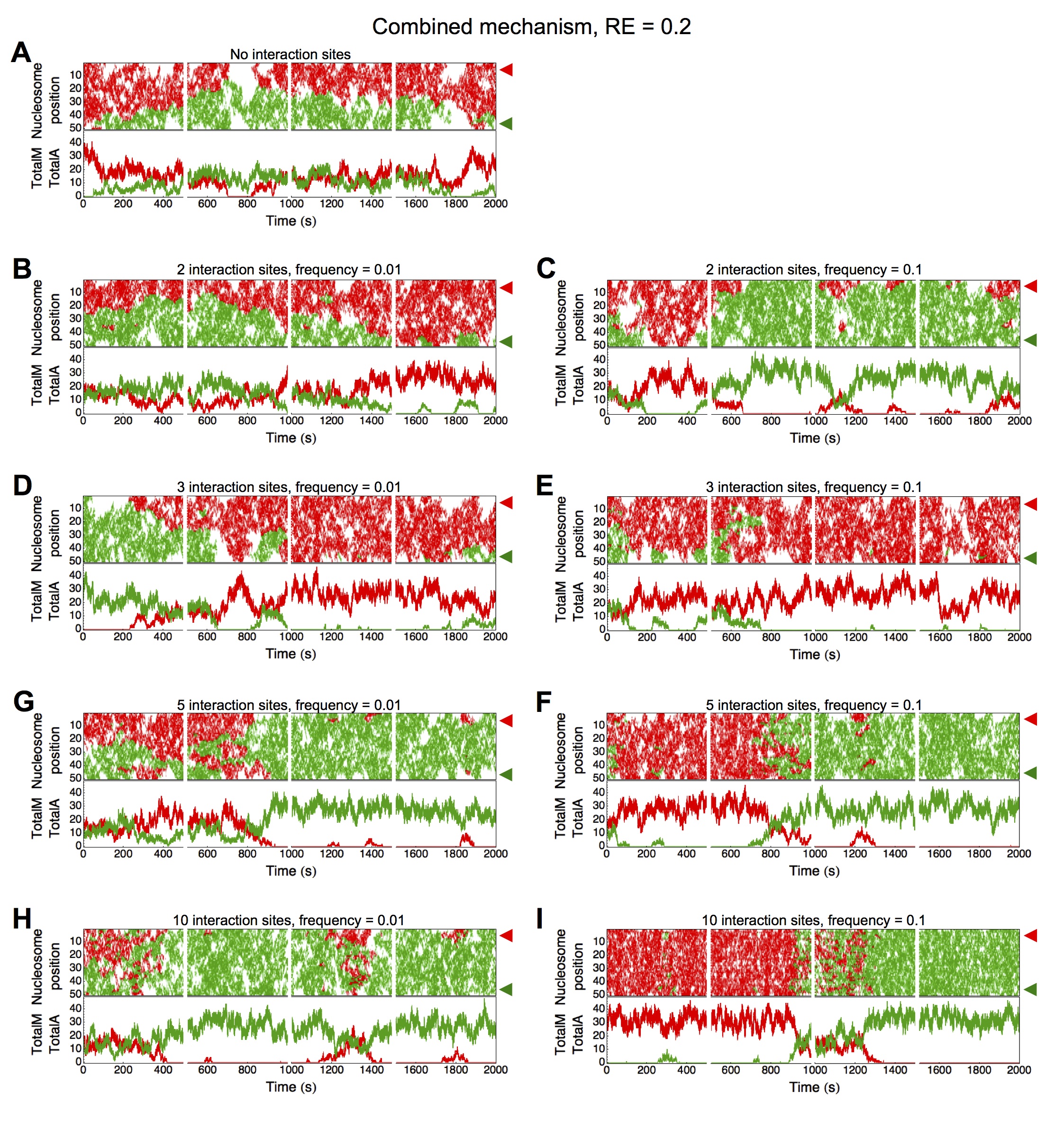

Supplement: Supplementary file 9 — Additional file 9: Figure S5: The influence of chromatin connectivity on the combined mechanism (RE =0.2), related to Figure 7. (A-I) The figure shows simulation of the combined mechanism with recruitment-efficiency 0.2 (krecruitment =0.48 s-1). Each subfigure shows a row of four simulations of 500 s each as an illustration of the model behavior. Top panels of each subfigure show the position (y-axis) of the methylation (red) and acetylation (green) over time (x-axis), initiation sites indicated by red and green arrowheads (on positions 5 and 45, respectively). Bottom panels show the total amount of each modification over time, corresponding to the top panel. Left column figures (B, D, F, H) show interaction at k interaction =0.01 s-1, right column figures (C, E, G, I) show interactions at k interaction =0.1 s-1. The other parameters used in these simulations are listed in Table 1. (A) Zero interaction sites. (B, C) Two interaction sites at positions 15 and 35. (D, E) Three interaction sites at positions 12, 25, and 38. (F, G) Five interaction sites at positions 8, 16, 25, 34, and 42. (H, I) Ten interaction sites at positions 3, 8, 13, 18, 23, 28, 33, 38, 43, and 48. (JPEG 2 MB) [file 13072_2014_336_MOESM9_ESM.jpeg]
