# Supplementary material for: Mechanistic stochastic model of histone modification pattern formation
Source: Epigenetics Chromatin. 2014 Oct 27;7:30. doi: 10.1186/1756-8935-7-30 (PMC4234852; doi:10.1186/1756-8935-7-30)
Supplement: Supplementary file 10 — Additional file 10: S5: Summarized list of model reactions. (DOCX 19 KB) [file 13072_2014_336_MOESM10_ESM.docx]

**Additional Information**

**S3. List of model reactions**

The following characters are used: M, U, A, and n for methylated, unmodified, acetylated, and ‘any’ nucleosome state. Subscripts *i* and *j* are any location on the array, i±1 is the left or right neighbor nucleosome however this is restricted between 1 and N total array length. Thus reactions across the model boundary do not exist. Superscript ‘bind’ and ‘loop’ represent a nucleosome on the binding/initiation sites and interaction sites respectively. Mt, At, and T represent methyltransferase, acetyltransferase, and either transferase, when T is placed between brackets the reaction is independent of transferase presence.

Initiation:

N^bind^ > TN^bind^

K_on_ *N^bind^

Enzyme modification:

MtU_i_ > MtM_i_

K_enzyme_ * MtU_i_

AtU_i_ > AtA_i_

K_enzyme_ * AtU_i_

If either enzyme is not explicitly modeled the modification reactions are

(Mt)U_i_ > (Mt)A_i_

Or

(At)U_i_ > (At)M_i_

K_modification_ * (T)U_i_

Demodification:

(T)M_i_ > (T)U_i_

(T)A_i_ > (T)U_i_

K_demodification_ * (T)N_i_

Release:

TN_i_ > N_i_

K_off_ * TN_i_

Sliding:

TN_i_ + N_i±1_ > N_i_ +TN_i±1_

K_slide_ * TN_i_ * N_i±1_

Recruitment:

M_i_ > MtM_i_

K_recruit_ * M_i_

A_i_ > AtA_i_

K_recruit_ * A_i_

Enzyme Neighbor modification:

MtN_i_ +(T)U_i±1_ > MtN_i_ +(T)M_i±1_

AtN_i_ +(T)U_i±1_ > AtN_i_ +(T)A_i±1_

K_neighbor-modification_ * TN_i_ +(T)U_i±1_

Looping:

N^loop^_i_ + TN^loop^_j_ > TN^loop^_i_ + N^loop^_j_

TN^loop^_i_ + N^loop^_j_ > N^loop^_i_ + TN^loop^_j_

AtN^loop^_i_ + MtN^loop^_j_ > MtN^loop^_i_ + AtN^loop^_j_

TN^loop^_i_ + TN^loop^_j_ > TN^loop^_i_ + TN^loop^_j_

N^loop^_i_ + N^loop^_j_ > N^loop^_i_ + N^loop^_j_

K_loop_ * (T)N^loop^_i_ * (T)N^loop^_j_

Initiation sites were always chosen at nucleosome position 25 for the one-enzyme models and at positions 5 and 45 (for At and Mt respectively) when modeled together. Interaction sites were chosen at equidistant sites from the border and each other.

| Number of interaction sites | Interaction site position |
| --- | --- |
| 1 | 15, 35 |
| 3 | 12, 25, 38 |
| 5 | 8, 16, 25, 34, 42 |
| 10 | 3, 8, 13, 18, 23, 28, 33, 38, 43, 48 |
